# Development of La_1.7_Ca_0.3_Ni_1−y_Cu_y_O_4+δ_ Materials for Oxygen Permeation Membranes and Cathodes for Intermediate-Temperature Solid Oxide Fuel Cells

**DOI:** 10.3390/membranes12121222

**Published:** 2022-12-02

**Authors:** Elena Filonova, Artem Gilev, Tatyana Maksimchuk, Nadezhda Pikalova, Kiryl Zakharchuk, Sergey Pikalov, Aleksey Yaremchenko, Elena Pikalova

**Affiliations:** 1 Department of Physical and Inorganic Chemistry, Institute of Natural Sciences and Mathematics, Ural Federal University, 620002 Yekaterinburg, Russia; 2 Laboratory of Chemical Design of New Multifunctional Materials, Institute of Natural Sciences and Mathematics, Ural Federal University, 620002 Yekaterinburg, Russia; 3 Institute of High Temperature Electrochemistry, Ural Branch of the Russian Academy of Sciences, 620137 Yekaterinburg, Russia; 4 Department of Chemical Materials Science, Institute of Natural Sciences and Mathematics, Ural Federal University, 620002 Yekaterinburg, Russia; 5 Institute of Metallurgy, Ural Branch of the Russian Academy of Sciences, 620016 Yekaterinburg, Russia; 6 CICECO—Aveiro Institute of Materials, Department of Materials and Ceramic Engineering, University of Aveiro, 3810-193 Aveiro, Portugal; 7 Department of Environmental Economics, Graduate School of Economics and Management, Ural Federal University, 620002 Yekaterinburg, Russia

**Keywords:** SOFCs, solid oxide fuel cells, cathodes, lanthanum nickelate, oxygen permeation membrane, electroconductivity, oxygen permeability, polarization resistance, electrode microstructure, collector layer

## Abstract

The La_1.7_Ca_0.3_Ni_1−y_Cu_y_O_4+δ_ (y = 0.0–0.4) nickelates, synthesized via a solid-state reaction method, are investigated as prospective materials for oxygen permeation membranes and IT-SOFC cathodes. The obtained oxides are single-phase and possess a tetragonal structure (*I4*/*mmm* sp. gr.). The unit cell parameter *c* and the cell volume increase with Cu-substitution. The interstitial oxygen content and total conductivity decrease with Cu-substitution. The low concentration of mobile interstitial oxygen ions results in a limited oxygen permeability of Cu-substituted La_1.7_Ca_0.3_NiO_4+δ_ ceramic membranes. However, increasing the Cu content over y = 0.2 induces two beneficial effects: enhancement of the electrochemical activity of the La_1.7_Ca_0.3_Ni_1−y_Cu_y_O_4+δ_ (y = 0.0; 0.2; 0.4) electrodes and decreasing the sintering temperature from 1200 °C to 900 °C. Enhanced electrode activity is due to better sintering properties of the developed materials ensuring excellent adhesion and facilitating the charge transfer at the electrode/electrolyte interface and, probably, faster oxygen exchange in Cu-rich materials. The polarization resistance of the La_1.7_Ca_0.3_Ni_1.6_Cu_0.4_O_4+δ_ electrode on the Ce_0.8_Sm_0.2_O_1.9_ electrolyte is as low as 0.15 Ω cm^2^ and 1.95 Ω cm^2^ at 850 °C and 700 °C in air, respectively. The results of the present work demonstrate that the developed La_1.7_Ca_0.3_Ni_0.6_Cu_0.4_O_4+δ_-based electrode can be considered as a potential cathode for intermediate-temperature solid oxide fuel cells.

## 1. Introduction

The growing demand for highly efficient, clean, and renewable energy sources requires increasingly intensive development of sustainable routes, including hydrogen [1,2], and alternative energy generation based on natural [3,4,5] and biofuels [6,7]. Hydrogen energy has an advantage among the listed technologies of the future since H_2_ is one of the common and abundant elements in nature and is a universal energy carrier [1,8].

Among various hydrogen-based energy production and conversion systems, solid oxide fuel cells (SOFCs) are recognized as one of the most promising technologies due to the numerous environmental and technical advantages they offer [9,10,11]. The main superiorities of SOFCs include high efficiency [12,13] and the ability to be used, along with hydrogen, with a wide variety of fuels such as hydrocarbons, natural gas, biogas, etc. [14,15,16].

However, the industrial commercialization of SOFCs is limited due to their performance reduction with time at high operating temperatures (800–1000 °C). This is a result of the degradation of SOFC structural parts [17,18], the main contribution to which is made by the chemical interaction between them [19]. Reducing the operating temperatures of SOFCs to 600–800 °C by selecting new functional materials would improve the durability of SOFCs and reduce their costs [14,20]. However, lowering the operating temperature leads to an increase in the activation energy of the oxygen reduction reaction (ORR) and an increase in the area-specific resistance of the electrodes (ASR), especially of the cathode. The use of oxide materials with mixed electron-ionic conductivity (MIEC) as cathodes can significantly improve surface oxygen exchange kinetics due to the expanded zone of electrochemical reactions, thus enabling the efficient operation of SOFCs at intermediate and low temperatures [21].

Due to the ambipolar conductivity, complex oxides with the Ruddlesden–Popper structure Ln_2_NiO_4+δ_ (Ln = La, Pr, Nd) and their substituted derivatives have been successfully tested as cathode materials either for intermediate-temperature fuel cells (IT-SOFCs) with oxygen-ion [22,23] and proton-conducting [24,25,26] electrolytes, or solid oxide electrolysis cells (SOEC) [24,27], as well as oxygen permeation membranes [28].

Among Ln_2_NiO_4+δ_ (Ln = La, Pr, Nd), it is the phase with Ln = La, which demonstrates the highest thermal stability (up to 1300 °C both in air and in atmospheres with a reduced oxygen content [29]). The literature data, summarized in Table 1, show that the substitutions into the La sublattice with divalent foreign cations such as alkaline earth ones (M = Ca^2+^, Sr^2+^, Ba^2+^) result in an increase in the total electrical conductivity of the La_2−x_M_x_NiO_4+δ_ oxides, which is an important functional property for both oxygen permeation membranes and air electrodes.

Although the polarization resistance of the corresponding La_2−x_M_x_NiO_4+δ_ electrodes often increases, it was shown by the methods of thermogravimetry and temperature-programmed isotope exchange [31,36,41,42], that the drop in the electrochemical activity of the La_2−x_M_x_NiO_4+δ_ electrodes with the increasing content of dopant was due to a decrease in the content of highly mobile interstitial oxygen.

The data in Table 1 show that among the lanthanum nickelates, substituted with alkaline earth cations, the highest electrochemical activity (the lowest polarization resistance, *R_p_*) of the corresponding electrodes in contact with a conventional oxygen-ion conducting Ce_0.8_Sm_0.2_O_1.9_ (SDC) electrolyte for IT-SOFCs is observed for the La_2−x_Ca_x_NiO_4+δ_ oxides. This fact stimulated a number of works aimed at the study of the electrical properties and electrochemical activity of the electrodes based on the Ca-doped La_2_NiO_4+δ_ derivatives obtained by the partial substitution of nickel by copper (Table 2). Simultaneous substitution by calcium into the La sublattice and by copper into the Ni sublattice was shown to have advantages in increasing the total conductivity of ceramics and improving the electrochemical activity of the corresponding electrodes in contact with the IT-SOFC electrolytes.

The data presented in Table 1 demonstrate that the La_2−x_Ca_x_NiO_4+δ_ electrodes with an oxide collector layer of LaNi_0.6_Fe_0.4_O_3−δ_ (LNF) exhibited better electrochemical activity than one-layer electrodes, as its use provided uniform current collection in the studied bilayer electrodes [34,35,36]. On the other hand, analysis of the characteristics of the previously studied Cu-doped La_2−x_Ca_x_NiO_4+δ_ electrodes in contact with a number of electrolytes for IT-SOFCs shown in Table 2 indicated that the La_1.7_Ca_0.3_Ni_1−y_Cu_y_O_4+δ_ electrodes in contact with the Ce_0.8_Gd_0.2_O_1.9_ solid electrolyte successfully operated without a collector layer [43].

**Table 2:** Total conductivity and electrode polarization resistance of La_2−x_Ca_x_NiO_4+δ_ nickelates at 700 °C on Cu content (y).

x	y	Composition	Ref.	σ, S cm^−1^	*R*_p_, Ω cm^2^	Electrolyte
0.0	0.1	La_2_Ni_0.9_Cu_0.1_O_4+δ_	[32]	58	0.16	BaCe_0.5_Zr_0.3_Dy_0.2_O_3−δ_
0.0	0.2	La_2_Ni_0.8_Cu_0.2_O_4+δ_	[32]	61	0.5	BaCe_0.5_Zr_0.3_Dy_0.2_O_3−δ_
0.0	0.2	La_2_Ni_0.8_Cu_0.2_O_4+δ_	[33]	28	4.95	LSGM
0.0	0.2	La_2_Ni_0.8_Cu_0.2_O_4+δ_	[44]	73	2.0	LSGM
0.0	0.2	La_2_Ni_0.8_Cu_0.2_O_4+δ_	[44]	73	3.2	La_10_Si_5_AlO_26.5_
0.0	0.3	La_2_Ni_0.7_Cu_0.3_O_4+δ_	[32]	55	1.2	BaCe_0.5_Zr_0.3_Dy_0.2_O_3−δ_
0.0	0.4	La_2_Ni_0.6_Cu_0.4_O_4+δ_	[33]	70	2.23	LSGM
0.3	0.1	La_1.7_Ca_0.3_Ni_0.9_Cu_0.1_O_4+δ_	[37]	130	0.89	LSGM
0.3	0.2	La_1.7_Ca_0.3_Ni_0.8_Cu_0.2_O_4+δ_	[37]	145	0.79	LSGM
0.3	0.3	La_1.7_Ca_0.3_Ni_0.7_Cu_0.3_O_4+δ_	[37]	156	0.50	LSGM
0.3	0.25	La_1.7_Ca_0.3_Ni_0.75_Cu_0.25_O_4+δ_	[45]		0.50	YSZ
0.3	0.25	La_1.7_Ca_0.3_Ni_0.75_Cu_0.25_O_4+δ_	[43]		0.07	Ce_0.8_Gd_0.2_O_1.9_
0.3	0.5	La_1.7_Ca_0.3_Ni_0.5_Cu_0.5_O_4+δ_	[45]		0.63	YSZ
0.3	0.75	La_1.7_Ca_0.3_Ni_0.25_Cu_0.75_O_4+δ_	[45]		1.95	YSZ
0.4	0.1	La_1.6_Ca_0.4_Ni_0.9_Cu_0.1_O_4+δ_	[46]	125		
0.4	0.2	La_1.6_Ca_0.4_Ni_0.8_Cu_0.2_O_4+δ_	[46]	103		
0.4	0.3	La_1.6_Ca_0.4_Ni_0.7_Cu_0.3_O_4+δ_	[46]	94		

In connection with the prospects of using the La_1.7_Ca_0.3_Ni_1−y_Cu_y_O_4+δ_ oxides as cathode materials for IT-SOFCs and materials for MIEC membranes, it is of interest to study the correlation between the features of the crystal structure, electrical transport properties in air and under various oxygen partial pressure values, oxygen permeability, as well as their thermomechanical properties and electrochemical activity of the La_1.7_Ca_0.3_Ni_1−y_Cu_y_O_4+δ_-based electrodes in contact with the SDC electrolyte without/with a collector layer.

## 2. Materials and Methods

### 2.1. Synthesis of the Materials and Preparation of the Compact Samples

The La_1.7_Ca_0.3_Ni_1−y_Cu_y_O_4+δ_ (y = 0.0–0.4) samples were synthesized via a conventional solid-state reaction method. Starting materials La_2_O_3_ (99.999% purity), CaCO_3_ (analytical grade, 99.4%), NiO, and CuO (analytical grade, 99.0%) were mixed and calcined at 1000 °C for 10 h. This was followed by milling in a planetary mill Fritsch Pulverizette 7 (Idar-Oberstein, Germany) with a rate of 250 rpm in isopropyl alcohol for 1 h in an agate drum with stainless steel milling media taken to the powder weight in the ratio of 5:1. Final synthesis was performed at 1200 °C for 10 h for the powders with y = 0.0–0.2 and at 1150 °C for 10 h for the powders with y = 0.3–0.4. The specific surface area of the powders was determined after the final milling for 1 h and drying by means of a META SORBI N.4.1 analyzer (Moscow, Russia) was in a range of 0.94–1.16 m^2^·g^−1^. The impurity content in the as-prepared powders caused by milling was determined by an inductively coupled plasma optical emission spectrometry (ICP-OES) using a Perkin Elmer OPTIMA 4300 DV device (Waltham, MA, USA) and amounted to approximately 0.3 (Fe) and 0.4 (Si) wt. % (Supplementary Data, Table S1). Additionally, impurity content was determined using SEM analysis, performed by means of a Tescan VEGA 3 scanning electron microscope equipped with an Ultim Max 40 detector (Brno – Kohoutovice, Czech Republic). The amount of impurities was in a range of 0.4–0.5 (Fe) and 0.4–0.7 (Si) wt. % (Supplementary Data, Table S1, Figures S1–S3).

For the preparation of dense ceramics, the synthesized La_1.7_Ca_0.3_Ni_1−y_Cu_y_O_4+δ_ (y = 0.0–0.4) powders were compacted uniaxially at 40 MPa into disk-shaped pellets (diameter 18 mm, thickness 1.5–2.0 mm). This was followed by isostatic pressing (200 Mpa) and annealing in air at various temperatures depending on Cu content (Table 3). The experimental density of the sintered ceramics (Table 3) was calculated from the sample weight and geometric dimensions of the polished disk-shaped samples. The rectangular bars for the dilatometric and electrical measurements were cut out of the disk-shaped samples using a Struers Minitom precision cutting machine (Copenhagen, Denmark) with a diamond cutoff disk.

### 2.2. X-ray Diffraction Analysis and Crystal Structure Refinement

The powder X-ray diffraction experiments, necessary for the determination of the phase purity of the La_1.7_Ca_0.3_Ni_1−y_Cu_y_O_4+δ_ (y = 0.0–0.4) samples and their crystal structure refinement, were performed using a Shimadzu XRD-7000 diffractometer (Tokyo, Japan) with a graphite monochromator in CuKα_1_ radiation at 25 °C in air. X-ray diffraction patterns were obtained over an angle range of 25 < 2θ° < 80 with a step of 0.02° and an exposition of 5 s per step. The structure refinement in the La_1.7_Ca_0.3_Ni_1−y_CuyO_4+δ_ series was performed via the Rietveld full-profile analysis method using the FullProf Suite program package [47].

The powder X-ray diffraction data, necessary to qualify the SDC and LNF phase purity, were obtained on a DRON 6 diffractometer (Saint Petersburg, Russia) with a graphite monochromator in CuKα_1_ radiation at 25 °C in air. X-ray patterns were collected over an angle range of 25 < 2θ° < 80 with a step of 0.04° and an exposition of 1 s per step.

### 2.3. Oxygen Nonstoichiometry Study

The room-temperature values of the absolute oxygen non-stoichiometry (δ) in the La_1.7_Ca_0.3_Ni_1−y_Cu_y_O_4+δ_ (y = 0.0–0.4) series were determined from the weight loss detected by the thermogravimetric analysis during the full reduction in a hydrogen-containing atmosphere. This is a traditional method employed for the studies of oxygen non-stoichiometry in oxides with easily reducible transition metal cations (e.g., Refs. [32,36]). The experiments were performed using a NETZSCH STA 449 F3 Jupiter thermal analyzer (Stuttgart, Germany) on heating in a 50% H_2_/50% Ar gas mixture from room temperature to 1000 °C at 10 °C min^−1^. The procedure resulted in a reduction of the complex oxide to a mixture of metallic nickel and copper co-existing with lanthanum and calcium oxides, as confirmed by the XRD analysis:

(1) $${\mathrm{La}}_{1.7}{\mathrm{Ca}}_{0.3}{\mathrm{Ni}}_{1-y}{\mathrm{Cu}}_{y}O_{4+\delta }+\left(1.15+\delta \right)H_{2}\to 0.85{\;\mathrm{La}}_{2}O_{3}+0.3\;\mathrm{CaO}+\left(1-y\right)\;\mathrm{Ni}+y\;\mathrm{Cu}+\left(1.15+\delta \right){\;H}_{2}O,$$

The value of δ in the initial nickelate was calculated from the change in the weight of the sample as follows:

(2) $$\delta =\left(\frac{m_{0}}{m_{\mathrm{red}}}M_{\mathrm{red}}-M_{\mathrm{cat}}\right)/M_{O}-4,$$

where m_0_ and m_red_ are the weight of the initial sample and the reduced sample, respectively, M_red_ is the molar mass of the reduced sample, M_cat_ is the molar mass of cations (taking the stoichiometry indexes into account), and M_O_ is the mass of one mole of oxygen atoms.

### 2.4. Scanning Electron Microscopy

Microstructural characterization of the sintered ceramic samples La_1.7_Ca_0.3_Ni_1−y_Cu_y_O_4+δ_ (y = 0.0; 0.2; 0.4) was performed using a Hitachi SU-70 microscope equipped with a Bruker Quantax 400 detector (Karlsruhe, Germany) for the energy dispersive spectroscopy (EDS) analysis. The microstructure and the chemical composition of the La_1.7_Ca_0.3_Ni_1−y_Cu_y_O_4+δ_ (y = 0.0; 0.2; 0.4) electrodes with/without the LaNi_0.6_Fe_0.4_O_3−δ_ (LNF) collector layer in contact with the SDC electrolyte substrate were examined using a Tescan VEGA 3 scanning electron microscope equipped with an Ultim Max 40 detector (Brno-Kohoutovice, Czech Republic).

### 2.5. Investigation of the Electrical Properties and Oxygen Permeability

The electrical conductivity of the La_1.7_Ca_0.3_Ni_1−y_Cu_y_O_4+δ_ (y = 0.0–0.4) ceramic samples was measured by a DC four-probe method as a function of temperature (100–950 °C) in air and as a function of oxygen partial pressure at 800 °C in the *pO_2_* range from ~10^−5^ to 1.0 atm. The platinum paste was applied onto the end-face surfaces of the rectangular bar-shaped samples to ensure uniform electrical contact. Pt wires were used as potential probes and current collectors. Oxygen partial pressure, *pO_2_*_,_ was regulated by the composition of the flowing O_2_ + N_2_ gas mixture and controlled using an electrochemical yttria-stabilized zirconia (YSZ) oxygen sensor. The flow rates of the gases were set by means of Bronkhorst mass flow controllers (Ruurlo, the Netherlands).

Investigation of oxygen permeability through the La_1.7_Ca_0.3_Ni_1−y_Cu_y_O_4+δ_ (y = 0.0; 0.4) ceramic membranes was performed at 800–950 °C using electrochemical YSZ solid electrolyte cells comprising an oxygen pump and a sensor [48]. The gas-tight ceramic disks of 1 mm in thickness were preliminarily tested for the absence of physical leakages under the total pressure gradient of ~2 atm (at 25 °C). Then, the disk was hermetically sealed onto the top of the YSZ electrochemical cell by a high-temperature glass sealant at 1100 °C. The oxygen partial pressure at the membrane feed side (*p_2_*) was equal to 0.21 atm, while the *pO_2_* at the permeate side (*p_1_*) varied in the course of measurements using the YSZ cell electrochemical pump and was controlled by the YSZ cell electrochemical sensor.

### 2.6. Dilatometric Studies

Thermal expansion of the La_1.7_Ca_0.3_Ni_1−y_Cu_y_O_4+δ_ (y = 0.0–0.4) dense ceramics was studied by dilatometry using a vertical Linseis L75 instrument (Selb, Germany). The measurements were performed in the heating/cooling modes at the rate of 3 °C·min^−1^ in flowing air between 25 and 1100 °C. The average thermal expansion coefficients (TECs) were calculated by linear approximation of the obtained dilatometric curves.

### 2.7. Electrode Polarization Resistance Experiments

The Ce_0.8_Sm_0.2_O_1.9_ (SDC) electrolyte substrates for the electrochemical measurements were fabricated from the powder obtained via a two-stage solid-state reaction route [34]. The material had a cubic fluorite-like structure (sp. gr. *Fm3m*) with a unit cell parameter *a* = 5.5453(6) Å. The powder was dry-pressed and sintered in air at 1620 °C for 5 h. The substrates had a relative density of 95–97% and the thickness of approximately 1 mm.

The electrode slurries were fabricated by mixing the La_1.7_Ca_0.3_Ni_1−y_Cu_y_O_4+δ_ (y = 0.0; 0.2; 0.4) electrode powders with ethanol and a 5 wt. % alcohol solution of the polyvinyl butyral binder with stirring for 2 h in an agate mortar. The electrodes were developed as single- and two-layer structures. To fabricate a single-layer electrode, the La_1.7_Ca_0.3_Ni_1-y_Cu_y_O_4+δ_ slurry was brush-painted symmetrically onto the opposite sides of the SDC substrates to produce the functional layers with a thickness of ~ 30 μm. The cells were sintered at 900–1100 °C for 2 h or at 1200 °C for 1 h. For the fabrication of two-layer electrodes, the LNF + 3 wt. % CuO current collector layer (thickness of 15, 30, or 50 μm) was deposited over the functional layer, pre-sintered at 1000 °C, and sintered at 900 °C for 2 h. The method of preparation of the current collector based on LNF with the CuO additive was developed earlier; the LNF powder was synthesized via a nitrate combustion method as described elsewhere [49]. The LNF had a rhombohedral perovskite structure (sp. gr. *R-3c*) with unit cell parameters *a* = 5.506(1); *c* = 13.252(1) Å.

The electrode performance was investigated by electrochemical impedance spectroscopy (EIS) on the fabricated symmetric cells using an electrochemical station comprising an SI-1260 frequency analyzer (Solartron Analytical, Farnborough, UK) and an SI-1287 electrochemical interface (Solartron Analytical, Farnborough, UK). The measurements were performed in a frequency range of 0.01 Hz–0.5 MHz with an applied *ac* signal amplitude of 30 mV. The EIS spectra were collected in the temperature range of 600–850 °C with a step of 50 °C in air. Each measurement was followed by recording the total *dc* cell resistance (*R_dc_*). the distribution functions of relaxation times (DFRTs) were obtained by Tikhonov regularization (TR) in the MATLAB version of the DRTtools software [50,51]. The calculations were carried out using a Gaussian-type function with the regularization parameter (RP) of 0.001. The combined Re-Im data setting was chosen in the calculation process.

## 3. Results and Discussion

### 3.1. Structure and Oxygen Content in the La_1.7_Ca_0.3_Ni_1−y_Cu_y_O_4+δ_ Materials

**Figure 1:**
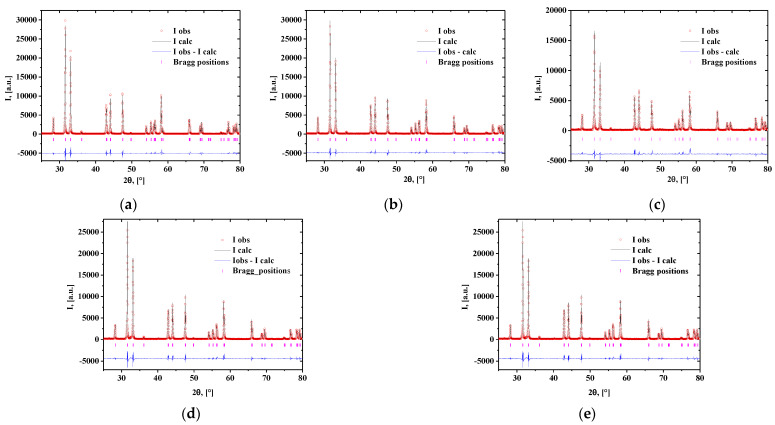
Observed (points) and calculated (lines) XRD patterns for the La_1.7_Ca_0.3_Ni_1−y_Cu_y_O_4+δ_ powdered samples: (**a**) La_1.7_Ca_0.3_NiO_4+δ_; (**b**) La_1.7_Ca_0.3_Ni_0.9_Cu_0.1_O_4+δ_; (**c**) La_1.7_Ca_0.3_Ni_0.8_Cu_0.2_O_4+δ_; (**d**) La_1.7_Ca_0.3_Ni_0.7_Cu_0.3_O_4+δ_; (**e**) La_1.7_Ca_0.3_Ni_0.6_Cu_0.4_O_4+δ_.

The XRD data for the powdered samples indicated the existence of a La_1.7_Ca_0.3_Ni_1−y_Cu_y_O_4+δ_ solid solution with a tetragonal structure. The results of the structural refinement of the XRD data obtained by a full-profile Rietveld analysis using the space group of *I4*/*mmm* are presented in Table 3. Crystal structure refinement was carried out according to the reference data for La_1.7_Ca_0.3_NiO_4+δ_ (JCPDS PDF#79-9864), assuming a uniform distribution of lanthanum/calcium and nickel/copper atoms in the corresponding sublattices. The observed and calculated XRD patterns are shown in Figure 1a–e.

As discussed in [52], the Goldschmidt tolerance factor, t, is a quantitative estimate of micro-strains in the Ln_2_NiO_4+δ_ structure due to a mismatch between the lattice parameters in the a,b-plane of the perovskite layers and the rock-salt-type layers [53]. The t values presented in Table 3 were calculated using the conventional equation presented elsewhere, for example in [24], and the data for ionic radii, reported in [54]. It was found that an increase in the copper content in the La_1.7_Ca_0.3_Ni_1−y_Cu_y_O_4+δ_ series resulted in a minor decrease in the t values. This means that a partial replacement of nickel ions by larger copper ions in the studied series causes an expansion along the (Ni/Cu)O2 layers, which, in turn, may increase the strain between the perovskite layers and the rock-salt-type layers.

**Figure 2:**
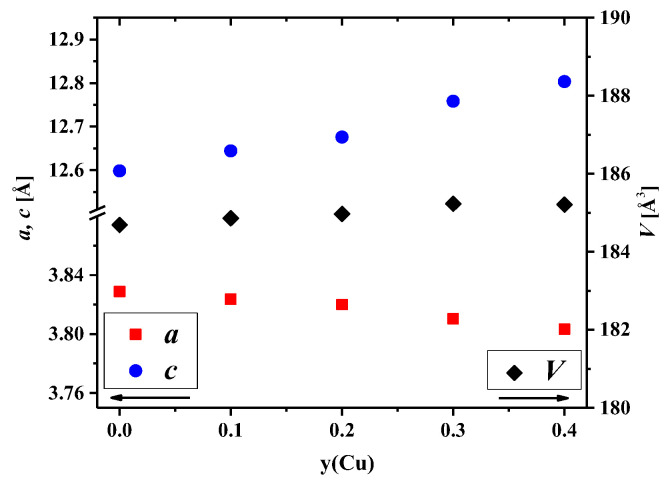
Dependencies of the unit cell parameters and volumes for the La_1.7_Ca_0.3_Ni_1−y_Cu_y_O_4+δ_ series on the Cu content.

Analysis of the concentration dependencies for the unit cell parameters and volumes presented in Figure 2 showed an increase in the *c* parameter and the unit cell volume. This was due to the difference in the ionic radii between nickel and copper cations [54]. However, the *a* parameter slightly decreased with an increase in the copper content. Similar behavior of the concentration dependencies of the unit cell parameters was observed earlier for La_2_Ni_1−y_Cu_y_O_4+δ_ [32,33], La_1.7_Ca_0.3_Ni_1−y_Cu_y_O_4+δ_ [37,45,46], Nd_2_Ni_1−y_Cu_y_O_4+δ_ [55], Nd_1.8_Sr_0.2_Ni_1−y_Cu_y_O_4+δ_ [56], Nd_1.6_Ca_0.4_Ni_1−y_Cu_y_O_4+δ_ [57], and Pr_1.7_Sr_0.3_Ni_1−y_Cu_y_O_4+δ_ [58].

**Figure 3:**
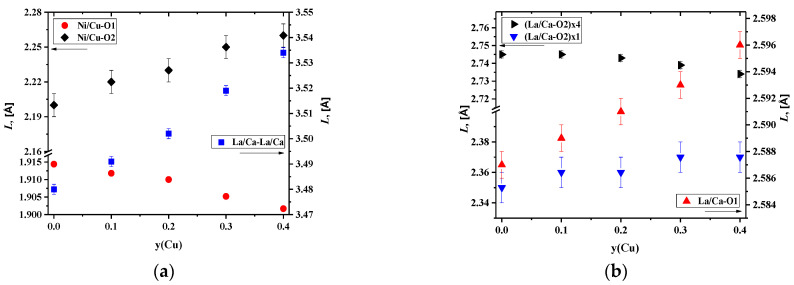
Interatomic distances in the La_1.7_Ca_0.3_Ni_1−y_Cu_y_O_4+δ_ series in dependence on the Cu content: (**a**) Ni/Cu–O, and (**b**) La/Ca–La/Ca, La/Ca–O.

The above-mentioned behavior of the concentration dependencies of the unit cell parameters for La_1.7_Ca_0.3_Ni_1−y_Cu_y_O_4+δ_ may be explained by the Jahn–Teller effect of copper cations [32,59]. Copper cations are able to form elongated CuO_6_ octahedrons in place of more regular NiO_6_ ones, as can be seen from the pictures presented in Figure 3a with the selected bond lengths calculated by the Rietveld method. The observed results can be explained by the preference of Cu^2+^ cations for a square planar coordination [54], resulting in elongated Ni/Cu–O2 bond length and shortened Ni/Cu–O1 bond length (Supplementary Materials, Figure S4).

The data on the selected bond lengths presented in Table 3 and Figure 3a,b illustrate that the *c* parameter, equal to the sum of the doubled (La/Ca–O2)×1, doubled Ni/Cu–O2, and La/Ca–La/Ca distances, increases due to an increase in all the interatomic distances mentioned. The room-temperature values of the oxygen non-stoichiometry, *δ*, for the La_1.7_Ca_0.3_Ni_1−y_Cu_y_O_4+δ_ samples, calculated from the thermogravimetric data, are given in Table 3. It was found that the incorporation of copper cations into the nickel sublattice resulted in a drop in the absolute oxygen content. This can be explained by the preference of copper cations for a lower average oxidation state in isostructural oxide compounds compared to nickel cations, as evident, for instance, from the data on oxygen non-stoichiometry of La_2_CuO_4+δ_ [60] and La_2_NiO_4+δ_ [61] under identical *pO_2_*-*T* conditions. The changes in the oxygen content correlate with the tendency of the copper cations to create structures with a square planar coordination [46] and agree well with the literature data for the Cu- substituted samples: La_2_Ni_1−y_Cu_y_O_4+δ_ [62], Nd_2_Ni_1−y_Cu_y_O_4+δ_ [58], La_1.6_Ca_0.4_Ni_1−y_Cu_y_O_4+δ_ [46], La_1.5_Pr_0.5_Ni_1−y_Cu_y_O_4+δ_ [63], Nd_1.5_Pr_0.5_Ni_1−y_Cu_y_O_4+δ_ [64], and Nd_1.6_Ca_0.4_Ni_1−y_Cu_y_O_4+δ_ [57].

### 3.2. Electrical Conductivity

**Figure 4:**
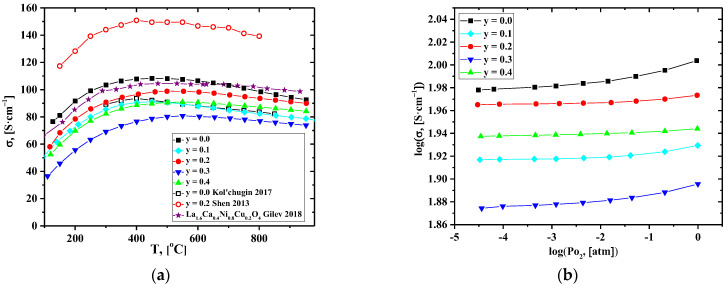
Dependencies of the total conductivity of the La_1.7_Ca_0.3_Ni_1−y_Cu_y_O_4+δ_ ceramics on: (**a**) on temperature, and (**b**) on *pO_2_* [35,37].

Figure 4a and Figure S5 show that the electrical conductivity of La_1.7_Ca_0.3_Ni_1−y_Cu_y_O_4+δ_ decreases with Cu content. The observed trend generally correlates with the literature data for the La_2_Ni_1−y_Cu_y_O_4+δ_ [32,33,59] and La_1.6_Ca_0.4_Ni_1−y_Cu_y_O_4+δ_ [46] series. Taking into account the changes in the oxygen content (Table 3), the decrease in conductivity may be assigned to the reduction in the concentration of the electronic-charge-carriers–electron-holes localized on the B-site transition metal cations (i.e., the concentration of Ni^3+^).

Shen et al. reported an increase in the total conductivity in the La_1.7_Ca_0.3_Ni_1−y_Cu_y_O_4+δ_ series [37]. The authors supposed that it could be related to increasing the content of the interstitial oxygen in the samples. However, according to our data on the concentration dependence of δ values, the interstitial oxygen content decreases with Cu-doping, at least at lower temperatures. A non-monotonous concentration dependence of the conductivity (Figure S5) and lower conductivity values compared to those presented in [37] may be related to the presence of the Si-containing impurities (most likely, in the form of inclusions of apatite-type La_10-x_Si_6_O_26+δ_-based phase [65]) in the samples as a result of contamination during the milling procedure (Figure S6). Additionally, it is worth noting that the La_1.7_Ca_0.3_Ni_1−y_Cu_y_O_4+δ_ samples in [37] were synthesized by a citrate–nitrate combustion method, and the sintering temperatures were lower than those used in the present study; thus, the formation of secondary phases could be less significant compared to the solid-state reaction method. Available literature reports, including our results [66,67,68] and the works of other authors [69,70,71,72], confirm that the preparative route can be responsible for variations in the physicochemical properties of the complex oxides. In the future, we plan to find a more advanced synthesis procedure for this series. Nevertheless, it should be noted that the conductivity values obtained in this study (103, 96, and 89 S cm^−1^ for y = 0.0, 0.2, and 0.4, correspondingly, at T = 700 °C) were close to those presented in [33,34,35,36,37,38,39,40,41,42,43,44,45,46] (Table 2).

Dependencies of the total conductivity on *pO*_2_ obtained for the La_1.7_Ca_0.3_Ni_1−y_Cu_y_O_4+δ_ compact samples are presented in Figure 4b. As for parent La_2_NiO_4+δ_, the conductivity slightly decreases with reducing *pO*_2_*,* which can be related to the reversible oxygen losses from the oxide structure and reduction of Ni cations. This process can be described as follows:

(3) $$2Ni_{Ni}^{•}+{O^{\prime \prime }}_{i}\overset{○\;○}{⇄}2Ni_{Ni}^{\times }+0.5O_{2}$$

The obtained data confirm that electronic transport in the studied materials is p-type. 

### 3.3. Oxygen Permeability and Ionic Transport

**Figure 5:**
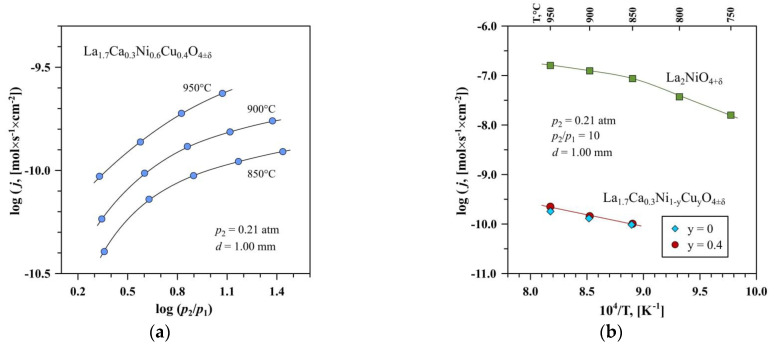
(**a**) Dependencies of the oxygen permeation fluxes through the La_1.7_Ca_0.3_Ni_0.6_Cu_0.4_O_4+δ_ ceramic membrane on the *pO_2_* gradient at 850–950 °C, and (**b**) Temperature dependences of the oxygen permeation fluxes through the La_1.7_Ca_0.3_Ni_0.6_Cu_0.4_O_4+δ_ and La_1.7_Ca_0.3_NiO_4+δ_ membranes under the fixed *pO_2_* gradient. The data on the undoped La_2_NiO_4+δ_ sample are taken from [65].

The oxygen permeability of the copper-free La_1.7_Ca_0.3_NiO_4±δ_ and La_1.7_Ca_0.3_Ni_0.6_Cu_0.4_O_4±δ_ ceramics with the highest copper content was studied at 850–950 °C. Figure 5a demonstrates the dependence of the oxygen permeation fluxes through the La_1.7_Ca_0.3_Ni_0.6_Cu_0.4_O_4±δ_ membrane on the oxygen partial pressure gradient in the studied temperature range, while Figure 5b compares the oxygen permeation fluxes through the undoped La_2_NiO_4+δ_ and La_1.7_Ca_0.3_Ni_1−y_Cu_y_O_4+δ_ membranes under the fixed oxygen partial pressure gradient across the membranes. The results indicate that the calcium-substituted and copper-co-substituted oxides exhibit nearly three orders of magnitude lower oxygen permeability compared to the parent lanthanum nickelate. This is accompanied by an increase in activation energy for oxygen permeation from 80 to 100 kJ/mol at 850–950 °C.

The suppression of oxygen ionic transport in La_1.7_Ca_0.3_Ni_1−y_Cu_y_O_4+δ_ should be attributed mainly to the decrease in the concentration of mobile oxygen ions caused by the acceptor-type substitution of La^3+^ by Ca^2+^. In particular, oxygen non-stoichiometry δ in La_1.7_Ca_0.3_Ni_1−y_Cu_y_O_4+δ_ corresponds to 0.01–0.05 at room temperature (Table 3) and is expected to tend to zero for both compositions at 850–950°C under atmospheric oxygen pressure, by analogy with La_2−x_Sr_x_NiO_4+δ_ (x = 0.2–0.4) nickelates [24,73]. As a result, oxygen permeability through the La_1.7_Ca_0.3_Ni_1−y_Cu_y_O_4+δ_ membranes is nearly independent on the copper content, within the limit of experimental uncertainty. On the contrary, oxygen excess in La_2_NiO_4+δ_ varies between δ = 0.15 at room temperature and ~0.08 at 950 °C [61,67]. Generally, the obtained results agree well with the literature data on the La_2−x_Ca_x_NiO_4+δ_ system indicating a decrease in oxygen diffusion and surface exchange coefficients with calcium doping and an increase in the corresponding activation energies [74,75].

One should also note that silica-based impurities may have negative effects on both bulk ionic transport and surface exchange kinetics. However, comparing the results on the oxygen permeability obtained in the present work with the data for silica-contaminated La_2_NiO_4+δ_ [65], one may conclude that the role of impurities is relatively insignificant compared to the effects induced by the acceptor-type substitution. Oxygen-ionic conductivity in mixed ionic-electronic conductors can be roughly estimated from the data on oxygen permeability and total electrical conductivity using the Wagner equation for the steady-state oxygen permeation flux density through the membrane bulk and neglecting surface exchange limitations (e.g., Ref. [67]). The calculations showed that the oxygen-ionic conductivity, σ_O_, in La_1.7_Ca_0.3_Ni_1−y_Cu_y_O_4+δ_ at 850–950 °C was six orders of magnitude lower than electronic conductivity. The estimated values of σ_O_ corresponded to 1.5×10^−4^ S/cm at 950 °C and decreased on cooling, being nearly independent of copper content.

### 3.4. Thermomechanical Properties

**Figure 6:**
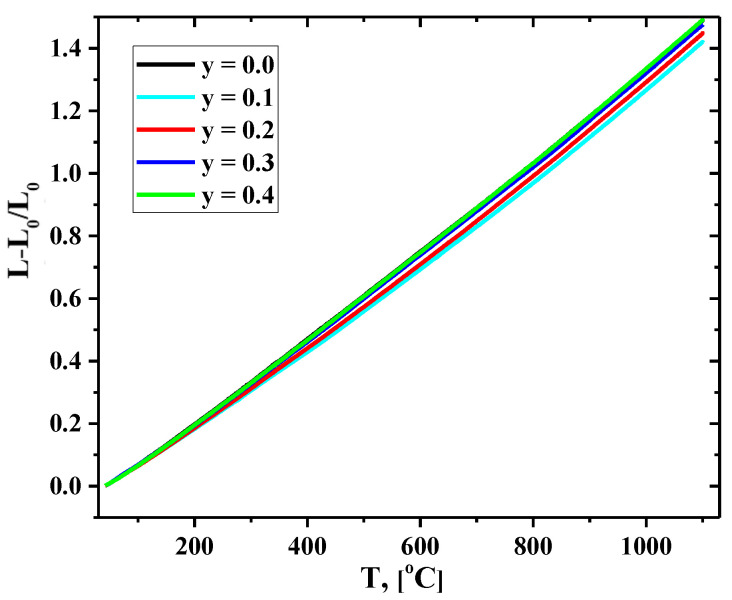
Temperature dependencies of the relative elongation of the La_1.7_Ca_0.3_Ni_1−y_Cu_y_O_4+δ_ ceramic samples collected in a heating mode.

Dilatometry experiments were performed both in heating and cooling modes. As an example, the temperature dependencies of the linear expansion of the La_1.7_Ca_0.3_Ni_1−y_Cu_y_O_4+δ_ compact samples collected in air during heating are shown in Figure 6. Based on the obtained experimental data, the averaged values of the La_1.7_Ca_0.3_Ni_1−y_Cu_y_O_4+δ_ linear thermal expansion coefficients (TECs) in the studied temperature range of 40–1100 °C were calculated and compared with the data presented in the literature (Table 4).

The average TECs of the La_1.7_Ca_0.3_Ni_1−y_Cu_y_O_4+δ_ ceramics at 40–1100 °C varied in a narrow range of (13.1–14.2)×10^−6^ K^−1^. For Cu-substituted compositions, a minor increase in the TEC value with an increase in the copper content seemed to correlate with the variations in the unit cell volume (Table 3). Noteworthy, the TEC values of the present study are in good agreement with those obtained in the previous investigations of thermal expansion of the Cu-substituted La_2−x_Ca_x_NiO_4+δ_ derivatives (Table 4). Additionally, the obtained data demonstrate that La_1.7_Ca_0.3_Ni_1−y_Cu_y_O_4+δ_ and Sm-doped ceria solid-state electrolytes (SDC), chosen for the electrochemical studies, are thermo-mechanically compatible.

### 3.5. Microstructure and Chemical Composition of the La_1.7_Ca_0.3_Ni_1−y_Cu_y_O_4+δ_-Based Cathodes

**Figure 7:**
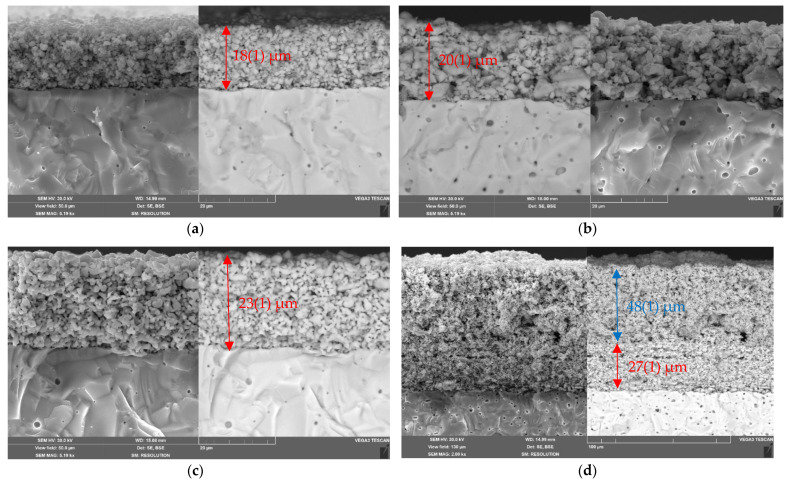
SEM images of the cross-sections of the La_1.7_Ca_0.3_Ni_1−y_Cu_y_O_4+δ_/SDC assemblies (Ts = 1000 °C): (**a**) single-layer electrode y = 0.0; (**b**) single-layer electrode y = 0.2; (**c**) single-layer electrode y = 0.4; (**d**) two-layer electrode y = 0.4 (Ts = 900 °C) with the LNF collector layer (50 μm, Ts = 900 °C). The thickness of the La_1.7_Ca_0.3_Ni_1−y_Cu_y_O_4+δ_ functional layers is shown in red, the thickness of the LNF collector is shown in blue.

The morphology of the La_1.7_Ca_0.3_Ni_0.6_Cu_0.4_O_4+δ_/SDC cathode layers sintered at 1000 °C was investigated using SEM. The corresponding micrographs presented in Figure 7a–c show that the resulting layers exhibit uniform grain distribution and high porosity. The SEM data also demonstrate that there are no cracks or delamination at the single-layer La_1.7_Ca_0.3_Ni_1−y_Cu_y_O_4+δ_/SDC and the two-layer LNF/La_1.7_Ca_0.3_Ni_1−y_Cu_y_O_4+δ_/SDC interfaces, as it can be seen from Figure 7c,d. After sintering, the thickness of the functional layers decreased and was in the range of 18–27 μm in dependence of the sintering temperature and sinterability of the electrode material. Despite the addition of a sintering additive (CuO), shrinkage of the collector layers sintered at 900 °C was less significant. As for example, the thickness of the LNF collector deposited with a thickness of 50 μm was evaluated from the SEM image as 47–48 μm. The average size of particles in the electrodes increased with increasing the copper content due to the better sintering ability of the Cu-containing materials (Figure S7). However, it varied within a narrow range of 0.4–1.1 μm.

The elemental distribution maps obtained by SEM/EDS for the La_1.7_Ca_0.3_Ni_1−y_Cu_y_O_4+δ_ (y = 0.0; 0.2; 0.4) electrode surface sintered at 1000 °C are shown in Figures S8–S10. The elemental compositions of the La_1.7_Ca_0.3_Ni_1−y_Cu_y_O_4+δ_ electrodes (Table S3) were found to be close to the nominal, within the accuracy of the EDS technique. The data show that the content of Si and Fe impurities in the sintered La_1.7_Ca_0.3_Ni_1−y_Cu_y_O_4+δ_ electrodes were in the range of 2.00–2.45 and 0.80–1.11 at. %, correspondingly. These values obtained for the impurities are higher than those (Table S2) obtained for the as-prepared materials, probably, due to additional mechanical treatments during the electrode slurry preparation. In addition, impurities of light-weight elements can diffuse towards the surface of the samples during sintering of the electrodes, which can also be a possible reason for their increased content on the surface of the electrodes.

### 3.6. Electrochemical Studies

**Figure 8:**
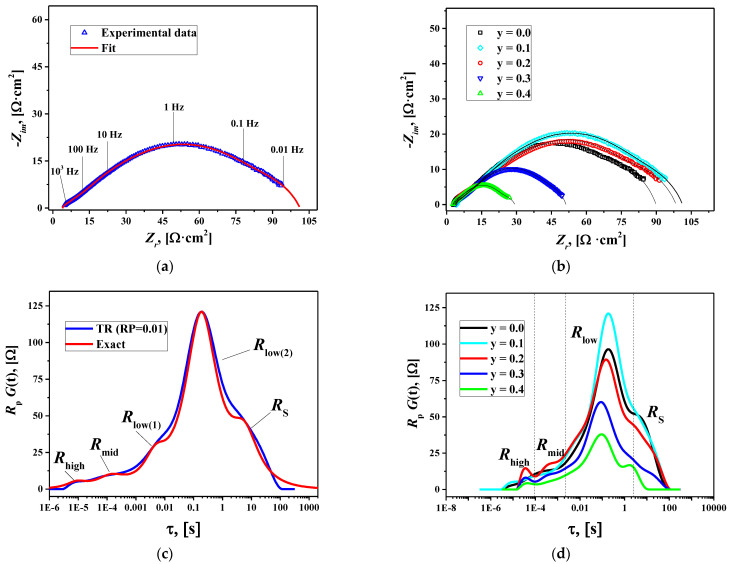
Fitted impedance spectra at 600 °C for the symmetrical cells: (**a**) La_1.7_Ca_0.3_Ni_0.9_Cu_0.1_O_4+δ_/SDC and (**b**) La_1.7_Ca_0.3_Ni_1−y_Cu_y_O_4+δ_/SDC; DFRTs at 600 °C for the symmetrical cells; (**c**) La_1.7_Ca_0.3_Ni_0.9_Cu_0.1_O_4+δ_/SDC, and (**d**) La_1.7_Ca_0.3_Ni_1−y_Cu_y_O_4+δ_/SDC.

The influence of the sintering temperature of the electrode layers on the polarization characteristics was evaluated in preliminary electrochemical experiments (Figure S11). It can be seen that the optimal sintering temperature decreases with Cu substitution by 300 °C for the sample with y = 0.4 (900 °C) compared to the copper-free La_1.7_Ca_0.3_NiO_4+δ_ electrode (1200 °C). The preliminary studies also revealed that the polarization resistance of the copper-substituted La_1.7_Ca_0.3_Ni_0.6_Cu_0.4_O_4+δ_ electrodes was lower than that of the parent material. To elucidate the impact of Cu substitution, a comprehensive analysis of the EIS data was performed. The impedance spectra for the La_1.7_Ca_0.3_Ni_1−y_Cu_y_O_4+δ_/SDC symmetrical cells (T_S_ = 1000 °C) collected at 600 °C are shown in Figure 8a,b. The complex shape of the spectrum in Figure 8a indicates the presence of several contributions to the overall polarization resistance (*R*_p_). A comparison of the impedance plots measured at 600 °C in Figure 8b demonstrates that the polarization resistance of the studied cells significantly decreases when y ≥ 0.3.

The distribution functions of relaxation times (DFRTs) for the obtained impedance data were calculated using Tikhonov regularization (TR) in DRTtools [50,51]. A Gaussian-type function and a regularization parameter (RP) of 0.01 were used for calculations. The obtained DFRTs are shown in Figure 8c,d. The curves demonstrate the presence of at least five contributions to the polarization resistance of the La_1.7_Ca_0.3_Ni_1−y_Cu_y_O_4+δ_/SDC cells. One can observe two relatively small peaks at high (*R*_high_) and middle (*R*_mid_) frequencies and three significantly larger overlapping peaks at low frequencies (*R*_low(1)_, *R*_low(2)_, and *R*_S_), which show that low-frequency processes dominate the polarization resistance of the studied cells. The *R*_low(1)_ and *R*_low(2)_ peaks merge into one peak (*R*_low_) at T > 650 °C. Consequently, the *R*_low(1)_ and *R*_low(2)_ resistances can be determined as distinct values only at lower temperatures (T = 600 °C). According to this, the equivalent circuit for fitting the impedance spectra consisted of ohmic resistance of the electrolyte *R_0_* and four (T ≥ 650 °C) or five (T = 600 °C) *R-CPE* elements connected in series. The *R-CPE* element, also known in the literature as ZARC, is a parallel combination of resistance and constant phase element. As an example, Figure 8a shows a satisfactory fit of the impedance spectrum for the La_1.7_Ca_0.3_Ni_0.9_Cu_0.1_O_4+δ_/SDC cell at 600 °C. The obtained fitting parameters were used to calculate the simulated DFRTs. For a ZARC, the simulated representation in τ-space is the following [78,79]:

(4) $$R\cdot G\left(\tau \right)=\frac{R}{2\pi }\cdot \frac{sin[\left(1-n\right)\pi ]}{cosh[nln(\tau /\tau_{0})]-cos[\left(1-n\right)\pi ]}$$

where $\tau_{0}=\sqrt[{n}]{R\cdot Q}$ is a time constant of the *R-CPE* element, while *Q* and n are the pseudo-capacitance and the associated exponent of the angular frequency in $Z_{CPE}=Q^{-1}{\left(j\omega \right)}^{-n}$, respectively.

The simulated DFRT can be obtained as a sum of individual contributions:

(5) $$R_{p}\cdot G\left(\tau \right)=\sum_{i}R_{i}\cdot G\left(\tau \right)$$

where $R_{p}=\sum_{i}R_{i}$.

**Figure 9:**
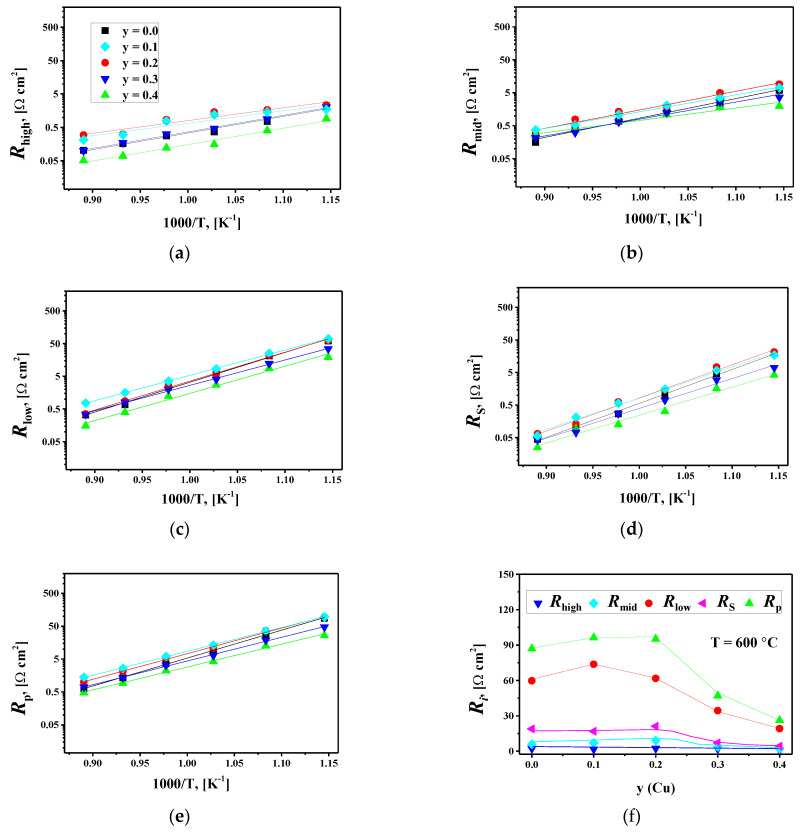
Partial contributions to the electrode polarization resistance and the total polarization resistance *R*_p_ on: (**a**–**e**) temperature, and (**f**) Cu content for the La_1.7_Ca_0.3_Ni_1−y_Cu_y_O_4+δ_/SDC symmetrical cells sintered at 1000 °C.

Figure 8c compares the simulated DFRT and the one calculated by the TR method for the La_1.7_Ca_0.3_Ni_0.9_Cu_0.1_O_4+δ_/SDC cell at 600 °C. The agreement between the two curves suggests the reliability of the fitting results. Similar results were observed for other studied cells. The resulting resistances *R*_high_, *R*_mid_, *R*_low_, *R*_S_, and *R*_p_ at different temperatures are presented in Figure 9.

For the identification of the processes characterized by the obtained resistances, the capacitances were calculated as follows [80]:

(6) $$C_{i}=R_{i}^{\left(\frac{1-n_{i}}{n_{i}}\right)}Q^{\frac{1}{n_{i}}}$$

In addition, activation energy (*E*_i_) for each process was determined from the slope of the corresponding log*R*_i_ = f(1/T) dependencies (Table 5).

The high-frequency and middle-frequency processes have capacitances near 10^−6^ and 10^−4^ F cm^−2^, respectively, and activation energies close to 1 eV, indicating that the *R*_high_ and *R*_mid_ resistances characterize interfacial charge transfer. Particularly, the *R*_high_ values can be associated with charge transfer through the electrolyte/electrode interface [81,82], while those of *R*_mid_ represent the charge transfer in the electrode or at the electrode/current collector interface [83,84,85]. For the low-frequency processes, the *C*_low(1)_, *C*_low(2)_, and *C*_S_ capacitances vary in ranges of 10^−4^–10^−3^, 1–4×10^−3^, and 10^−1^–1 F cm^−2^, respectively. These values are higher than typical double-layer capacitances and can be identified as “chemical” capacitances associated with changes in oxygen non-stoichiometry in the mixed conductor [81,86].

Taking into account the obtained capacitances and *E*_low_ activation energies, one can suggest that *R*_low_ resistances describe oxygen solid-state diffusion (*R*_low(1)_) and surface oxygen exchange (*R*_low(2)_) in the electrode material [81,84,85]. Larger *C*_S_ capacitances up to 1 F cm^−2^ and even lower frequencies for the *R*_S_ contribution compared to those for *R*_low_ imply that the former is related to the oxygen gas-phase diffusion in the pores of the electrodes [86]. However, significant activation energy (*E*_S_) suggests that the *R*_S_ resistances can be associated with a more complex process that includes not only oxygen gas-phase diffusion but also oxygen exchange at the electrode/gas-phase interface.

A comparison of the obtained resistances in Figure 9 shows that the *R*_low_=*R*_low(1)_+*R*_low(2)_ resistances (i.e., solid-state diffusion and surface exchange) make the most significant contribution to the total polarization resistance of the studied cells in the temperature range of 600–800 °C. However, the relative contribution of charge transfer processes (*R*_high_ and *R*_mid_) to the resulting *R*_p_ values noticeably increases at higher temperatures, and for some studied cells, it becomes predominant at 850 °C.

The increase in copper content in La_1.7_Ca_0.3_Ni_1−y_Cu_y_O_4+δ_ up to y = 0.2 has little effect on the polarization resistance of the studied cells, as seen in Figure 9f. At y ≥ 0.3, the *R*_low_ and *R*_S_ values decrease with increasing y leading to a reduction in the total polarization resistance. According to the results of the oxygen permeation study (Section 3.3), the Cu-doping shows an insignificant influence on the level of ionic conductivity, which is approximately the same for the first (x = 0.0) and the last (x = 0.4) member of the series. Boehm et al. reported that oxygen diffusion coefficients slightly decreased with copper doping in La_2_Ni_1-x_Cu_x_O_4+δ_ while the concentration of interstitial oxygen gradually decreased from 0.16 at x = 0 down to 0.01 at x = 1 [62]. However, copper substitution can facilitate the surface oxygen exchange in La_1.7_Ca_0.3_Ni_1−y_Cu_y_O_4+δ_, probably due to a decrease in oxygen excess from 0.05(1) to 0.00(1) (Table 3) and possible formation of oxygen vacancies in the perovskite layers at increased temperatures [86]. Conforming to [86], the rate of surface oxygen exchange in La_2_NiO_4+δ_ and La_2−x_Sr_x_NiO_4+δ_ is limited by incorporation due to the limited availability of oxygen vacancies. The authors disregarded the direct incorporation of the adsorbed oxygen into the interstitial site and suggested that the charged ion first moves into an apical oxygen vacancy and only after that moves into the interstitial site. Within this concept, the formation of new vacancies at the surface of La_1.7_Ca_0.3_Ni_1−y_Cu_y_O_4+δ_ with copper substitution, especially for highly doped samples, should promote surface exchange.

**Figure 10:**
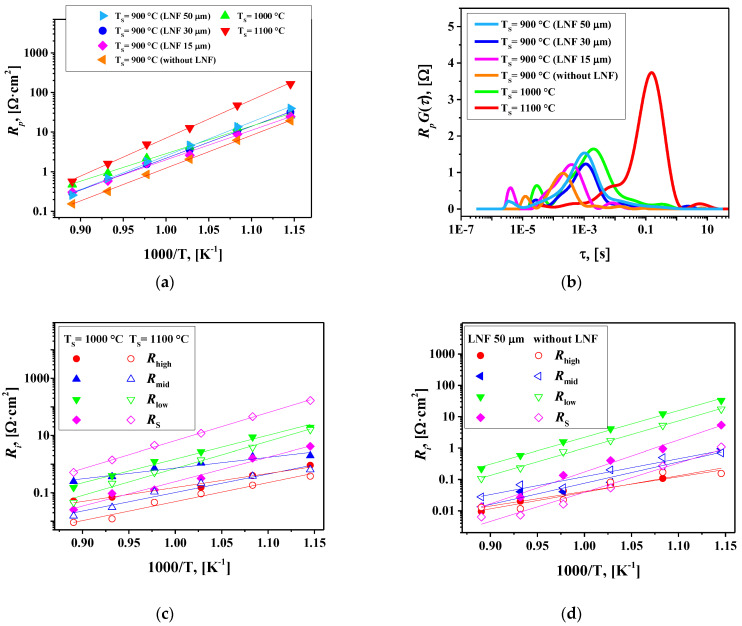
(**a**) Electrode polarization resistance versus temperature for different La_1.7_Ca_0.3_Ni_0.6_Cu_0.4_O_4+δ_/SDC cells; (**b**) DFRTs calculated by the regularization method at 800 °C; (**c**) partial contributions to *R*_p_ for the La_1.7_Ca_0.3_Ni_0.6_Cu_0.4_O_4+δ_/SDC cells fabricated at 1000 °C and 1100 °C; (**d**) partial contributions to *R*_p_ for the La_1.7_Ca_0.3_Ni_0.6_Cu_0.4_O_4+δ_/SDC cells fabricated at 900 °C with and without the LNF collector layer.

In this way, the increase in the *R*_low_ values (and, consequently, *R*_p_) with copper doping in the range of 0 ≤ y ≤ 0.2 can be due to slower oxygen-ion transport due to decreasing the interstitial oxygen content, while the reduction in the *R*_low_ values at y > 0.2 can be attributed to the facilitated surface exchange kinetics in these materials due to appearance of oxygen vacancies at the electrode operating temperatures. The microstructural factors and sinterability of the electrodes should also be taken into account. On the one hand, the electrodes with y = 0.3, 0.4 present a more homogeneous microstructure compared to that for the oxides with 0 ≤ y ≤ 0.2, while the gradual increase in grain size with copper doping of La_1.7_Ca_0.3_Ni_1−y_Cu_y_O_4+δ_ indicates better sinterability and, consequently, better grain connectivity (Figure S4), which should improve the performance. On the other hand, the increased particle size (and the decreased specific surface area) at a higher copper content can deteriorate the electrode performance as is discussed further in the paper. The latter could be a more critical parameter since the reduction in sintering temperature significantly decreases the polarization resistance of La_1.7_Ca_0.3_Ni_0.6_Cu_0.4_O_4+δ_ (see the next paragraph, Figure 10a and Figure S8). Hence, the microstructural factors should have a detrimental effect on the overall electrode performance. Still, the polarization resistance of La_1.7_Ca_0.3_Ni_1−y_Cu_y_O_4+δ_ decreases when y > 0.2 indicating that copper doping improves electrode performance despite the changes in microstructure, most probably, due to the enhanced surface oxygen exchange, as we discussed above. Nevertheless, more comprehensive research on the effect of the electrode microstructure on the polarization resistance, as well as studies on the bulk oxygen diffusion and surface oxygen exchange kinetics for La_1.7_Ca_0.3_Ni_1−y_Cu_y_O_4+δ_ are necessary.

For further optimization of the La_1.7_Ca_0.3_Ni_0.6_Cu_0.4_O_4+δ_/SDC symmetrical cell, the effect of sintering temperature (T_S_) and the role of the LaNi_0.6_Fe_0.4_O_3-δ_ (LNF) current collector were analyzed. The polarization resistance as a function of temperature for the La_1.7_Ca_0.3_Ni_0.6_Cu_0.4_O_4+δ_/SDC cells sintered at 900, 1000, and 1100 °C is shown in Figure 10a. One can see that the *R*_p_ values decrease when the sintering temperature is reduced from 1000 to 900 °C and significantly increase with the increase in T_S_ up to 1100 °C. This shows that T_S_ = 900 °C is the optimal sintering temperature for the studied cells. The DFRTs calculated by the regularization method (as described above) and the resistances of separate contributions are shown in Figure 10b,c, respectively. The increase in T_S_ led to reduced *R*_high_ and *R*_mid_ resistances, i.e., it facilitated charge transfer processes.

Higher sintering temperatures should improve interfacial connectivity between the cell components promoting charge transfer. At the same time, the value of the low-frequency contribution *R*_S_ significantly increases with increasing T_S_. This can be caused by the reduction in the specific surface area of the electrodes (an increase in the grain size), which leads to a decrease in the porosity of the electrode layers creating limitations for oxygen gas-phase diffusion toward the reaction sites. The reduced specific surface area could also limit the number of available sites for oxygen adsorption, slowing down the oxygen exchange process.

The reduction in the thickness of the LNF collector from 50 to 30 and then to 15 µm in the cells sintered at 900 °C leads to a slight decrease in polarization resistance at T < 750 °C, Figure 10a. Moreover, the La_1.7_Ca_0.3_Ni_0.6_Cu_0.4_O_4+δ_/SDC cell without the LNF collector shows the lowest polarization resistance, 1.95 Ω cm^2^ at 700 °C, compared to that of similar cells (T_S_ = 900 °C) with the current collector layer. The analysis of separate contributions to the total polarization resistance, Figure 10d, shows that adding LNF as a collector increases the *R*_low_ and especially *R*_S_ values. Assuming that the *R*_S_ resistances are associated with oxygen gas-phase diffusion followed by surface exchange, one can conclude that the LNF layer hampers gas-phase oxygen transport to the electrode surface and induces limitations for surface exchange, thus increasing the *R*_low_ values.

It was shown earlier that the application of the LNF collector onto the copper-free La_1.7_Ca_0.3_NiO_4+δ_ electrode led to a substantial decrease in the polarization resistance from 12.0 to 4.0 Ω cm^2^ at 700 °C (Table 1, Refs. [35,36]). However, one should note that the latter value is remarkably higher than *R*_p_ obtained for the single-layer La_1.7_Ca_0.3_Ni_0.6_Cu_0.4_O_4+δ_ electrode in the present work under similar conditions. Thus, the copper substitution of La_1.7_Ca_0.3_Ni_1−y_Cu_y_O_4+δ_ not only improves the overall electrochemical performance but also eliminates the need for an additional current collector layer. Summarizing, the increase in copper content in La_1.7_Ca_0.3_Ni_1−y_Cu_y_O_4+δ_ up to y = 0.4 reduces the optimal sintering temperature of electrodes of the SDC-based symmetric cells down to 900 °C and minimizes the polarization resistance down to 0.15, 0.31, 0.85 and 1.95 Ω cm^2^ at 850, 800, 750 and 700 °C, respectively.

## 4. Conclusions

In this study, the physicochemical properties and electrochemical performance of the La_1.7_Ca_0.3_Ni_1−y_Cu_y_O_4+δ_ (y = 0.0–0.4) nickelates as potential candidates for the application in the intermediate-temperature solid oxide fuel cells were investigated. The La_1.7_Ca_0.3_Ni_1−y_Cu_y_O_4+δ_ (y = 0.0–0.4) solid solutions synthesized via the conventional solid-state reaction method had a tetragonal structure (space group *I4*/*mmm*). The unit cell parameter *c* and cell volume increased in the La_1.7_Ca_0.3_Ni_1−y_Cu_y_O_4+δ_ series consistently with the larger size of Cu and with the concomitant decrease of the oxygen content in the lattice resulted in a moderate decrease in the total electrical conductivity value from 103 S cm^−1^ (y = 0.0) to 91 S cm^−1^ (y = 0.4) at 700 °C. The low concentration of the interstitial oxygen ions in the lattice as a result of the acceptor-type substitution with calcium resulted in a limited oxygen permeability of the Cu-substituted La_1.7_Ca_0.3_NiO_4+δ_ membranes compared to the undoped lanthanum nickelate. Cu substitution was shown to have no visible influence on the oxygen permeability. Moreover, the high level of Cu substitution (y > 0.2) allowed the reduction in the polarization resistance of the La_1.7_Ca_0.3_Ni_1.6_Cu_0.4_O_4+δ_ electrodes in contact with Ce_0.8_Sm_0.2_O_1.9_ solid electrolyte from 0.6 Ω cm^2^ (y = 0.0) down to 0.15 Ω cm^2^ (y = 0.4) at 850 °C in air. This was accompanied by a decrease in the activation energy of the electrode process from 1.68 to 1.38 eV. The electrode performance enhancement can be associated with facilitated surface exchange kinetics, as well as improvements in powder sintering and, accordingly, contact/adhesion both between the electrode and electrolyte and between grains.

The optimum sintering temperature of porous electrode layers was found to reduce from 1200 °C for y = 0.0 to 900 °C for y = 0.4. In contrast to the copper-free La_1.7_Ca_0.3_NiO_4+δ_ electrodes, the application of the LaNi_0.6_Fe_0.4_O_3-δ_ collector layer on the La_1.7_Ca_0.3_Ni_1.6_Cu_0.4_O_4+δ_ electrode had no positive impact on the electrochemical activity. The results of the present work imply that the designed single-layer La_1.7_Ca_0.3_Ni_0.6_Cu_0.4_O_4+δ_ electrodes can be recommended for the application as cathodes in IT-SOFCs. The electrical properties and electrode performance can be further optimized using chemical techniques for the synthesis of fine La_1.7_Ca_0.3_Ni_1−y_Cu_y_O_4+δ_ powders without ball milling and, consequently, excluding Si and Fe contaminations.

## Figures and Tables

**Table 1:** Total conductivity and electrode polarization resistance of La_2−x_M_x_NiO_4+δ_ (M = Ca, Sr, Ba) nickelates at 700 °C.

M	x	Composition	Ref.	σ, S cm^−1^	*R*_p_, Ω cm^2^	Electrolyte/Collector
-	0.0	La_2_NiO_4+δ_	[30]	65		
-	0.0	La_2_NiO_4+δ_	[31]	56	0.73	Ce_0.8_Sm_0.2_O_1.9_/LNF
-	0.0	La_2_NiO_4+δ_	[32]	76	0.10	BaCe_0.5_Zr_0.3_Dy_0.2_O_3−δ_
-	0.0	La_2_NiO_4+δ_	[33]	53	5.75	LSGM
Ca	0.1	La_1.9_Ca_0.1_NiO_4+δ_	[34]	67	1.78	Ce_0.8_Sm_0.2_O_1.9_/LNF
Ca	0.3	La_1.7_Ca_0.3_NiO_4+δ_	[35]	86	12.4	Ce_0.8_Sm_0.2_O_1.9_
Ca	0.3	La_1.7_Ca_0.3_NiO_4+δ_	[36]	86	4.01	Ce_0.8_Sm_0.2_O_1.9_/LNF
Ca	0.3	La_1.7_Ca_0.3_NiO_4+δ_	[37]		1.4	LSGM
Ca	0.5	La_1.5_Ca_0.5_NiO_4+δ_	[38]	150	0.061	BaZr_0.1_Ce_0.7_Y_0.2_O_3-*δ*_
Sr	0.3	La_1.7_Sr_0.3_NiO_4+δ_	[35]	69	12.8	Ce_0.8_Sm_0.2_O_1.9_
Sr	0.5	La_1.5_Sr_0.5_NiO_4+δ_	[38]	140	0.128	BaZr_0.1_Ce_0.7_Y_0.2_O_3-*δ*_
Sr	0.75	La_1.25_Sr_0.75_NiO_4+δ_	[30]	253		
Sr	1.6	La_0.4_Sr_1.6_NiO_4+δ_	[39]	224	7.9	Ce_0.9_Gd_0.1_O_2-δ_
Ba	0.05	La_1.95_Ba_0.05_NiO_4+δ_	[40]	73	1.11	BaCe_0.5_Zr_0.3_Dy_0.2_O_3−δ_
Ba	0.3	La_1.7_Ba_0.3_NiO_4+δ_	[35]	70	16.1	Ce_0.8_Sm_0.2_O_1.9_
Ba	0.5	La_1.5_Ba_0.5_NiO_4+δ_	[38]	124	0.127	BaZr_0.1_Ce_0.7_Y_0.2_O_3-*δ*_

**Table 3:** Tolerance factors, refined structural parameters, and absolute oxygen non-stoichiometry values for the La_1.7_Ca_0.3_Ni_1−y_Cu_y_O_4+δ_ series.

y		0.0	0.1	0.2	0.3	0.4
***t***		0.895	0.893	0.891	0.890	0.888
***a*, Å**		3.829(1)	3.824(1)	3.820(1)	3.810(1)	3.803(1)
***c*, Å**		12.598(1)	12.644(1)	12.676(1)	12.758(1)	12.804(1)
***V,* Å^3^**		184.689(9)	184.863(9)	184.975(9)	185.236(9)	185.206(7)
**ρ_XRD_, g** **·cm^−3^**		6.682	6.673	6.678	6.677	6.690
**z(La/Ca)**		0.362(1)	0.362(1)	0.362(1)	0.362(1)	0.362(1)
**z(O2)**		0.174(1)	0.176(1)	0.176(1)	0.177(1)	0.177(1)
***L*, Å**	**La/Ca-La/Ca**	3.480(2)	3.491(2)	3.502(2)	3.519(2)	3.534(2)
	**Ni/Cu-La/Ca**	3.218(1)	3.218(1)	3.219(1)	3.219(1)	3.218(1)
	**Ni/Cu-Ni/Cu**	3.829(1)	3.824(1)	3.820(1)	3.810(1)	3.803(1)
	**Ni/Cu-O1x4**	1.914(1)	1.912(1)	1.909(1)	1.905(1)	1.902(1)
	**Ni/Cu-O2x2**	2.202(9)	2.219(9)	2.234(9)	2.249(8)	2.261(8)
	**La/Ca-O1x4**	2.587(1)	2.589(1)	2.591(1)	2.593(1)	2.596(1)
	**La/Ca-O2x4**	2.745(2)	2.745(2)	2.743(2)	2.739(2)	2.734(2)
	**La/Ca-O2x1**	2.350(9)	2.357(9)	2.361(9)	2.371(8)	2.374(8)
**R_Br_**		2.88	2.63	6.17	2.57	3.94
**R_f_**		2.39	1.98	3.79	1.62	2.10
**R_exp_**		4.27	4.28	4.82	5.36	5.35
**R_wp_**		10.5	10.00	11.6	10.1	10.1
**R_p_**		8.03	7.28	8.80	7.78	7.79
***χ* ^2^**		6.01	5.55	5.79	3.53	3.56
**δ_TGA_**		0.05(1)	0.01(1)	0.01(1)	0.00(1)	0.01(1)
**T_sintering_, °C**		1450	1415	1380	1340	1300
**ρ, g cm^−3^**		6.44	6.52	6.57	6.39	6.51
**Relative density, %**		96.4	97.7	98.3	95.7	97.3

**Table 4:** Average TEC values for the La_1.7_Ca_0.3_Ni_1−y_Cu_y_O_4+δ_ ceramics and other Cu- and Ca-substituted La_2_NiO_4+δ_ derivatives.

Sample	Ref.	T Range, °C	TEC × 10^6^, K^−1^		
			Heating	Average Value	
				Heating	Cooling
**y = 0.0**	This work	40–300	13.0(1)		
		300–800	14.0(1)		
		800–1100	15.2(1)		
		40–1100		14.1(1)	13.9(1)
**y = 0.1**	This work	40–300	11.8(1)		
		300–800	13.3(1)		
		800–1100	15.1(1)		
		40–1100		13.4(1)	13.1(1)
**y = 0.2**	This work	40–300	12.2(1)		
		300–800	13.6(1)		
		800–1100	15.2(1)		
		40–1100		13.7(1)	13.4(1)
**y = 0.3**	This work	40–300	12.7(1)		
		300–800	13.9(1)		
		800–1100	15.2(1)		
		40–1100		14.0(1)	13.8(1)
**y = 0.4**	This work	40–300	12.9(1)		
		300–800	14.1(1)		
		800–1100	15.3(1)		
		40–1100		14.2(1)	13.9(1)
La_2_Ni_0.8_Cu_0.2_O_4−δ_	[33]	50–1000	13.9		
La_2_Ni_0.8_Cu_0.2_O_4−δ_	[76]	100–980	14.2		
La_2_Ni_0.6_Cu_0.4_O_4−δ_	[33]	50–1000	13.0		
La_1.7_Ca_0.3_Ni_0.75_Cu_0.25_O_4−δ_	[45]	50–850	14.4		
La_1.6_Ca_0.4_Ni_0.9_Cu_0.1_O_4+δ_	[46]	25–1000	14.9		
SDC	[77]	350–900	12.3		

**Table 5:** Activation energies of the processes that contribute to the polarization resistance of the La_1.7_Ca_0.3_Ni_1−y_Cu_y_O_4+δ_/SDC symmetrical cells.

y	*E*_high_, eV	*E*_mid_, eV	*E*_low_, eV	*E*_S_, eV	*E*_p_, eV
**0.0**	0.99 ± 0.07	1.17 ± 0.08	1.81 ± 0.06	2.07 ± 0.05	1.68 ± 0.02
**0.1**	0.74 ± 0.1	1.02 ± 0.04	1.54 ± 0.01	1.90 ± 0.08	1.44 ± 0.01
**0.2**	0.76 ± 0.1	1.12 ± 0.08	1.76 ± 0.05	2.02 ± 0.09	1.55 ± 0.04
**0.3**	0.98 ± 0.03	1.02 ± 0.07	1.54 ± 0.04	1.82 ± 0.09	1.43 ± 0.03
**0.4**	0.97 ± 0.07	0.75 ± 0.09	1.65 ± 0.09	1.70 ± 0.1	1.38 ± 0.05

## Data Availability

Not applicable.

## Supplementary Materials

The following supporting information can be downloaded at: https://www.mdpi.com/article/10.3390/membranes12121222/s1, Figure S1: SEM/EDS elemental distribution maps of the La_1.7_Ca_0.3_NiO_4+δ_ powder; Figure S2: SEM/EDS elemental distribution maps of the La_1.7_Ca_0.3_Ni_0.8_Cu_0.2_O_4+δ_ powder; Figure S3: SEM/EDS elemental distribution maps of the La_1.7_Ca_0.3_Ni_0.6_Cu_0.4_O_4+δ_ powder; Figure S4: Crystal structure of La_1.7_Ca_0.3_Ni_1−y_Cu_y_O_4+δ_; Figure S5: Concentration dependencies of the total conductivity of the La_1.7_Ca_0.3_Ni_1y_Cu_y_O_4+δ_ compact samples collected in air; Figure S6: SEM/EDS: fractured surface of La_1.7_Ca_0.3_Ni_0.8_Cu_0.2_O_4+δ_ ceramics sintered at 1380 °C; Figure S7: Particles size distribution in the La_1.7_Ca_0.3_Ni_1−y_Cu_y_O_4+δ_ electrodes based on the SEM data; Figure S8: SEM/EDS elemental distribution maps of the La_1.7_Ca_0.3_NiO_4+δ_ electrode surface (T_S_ = 1000 °C); Figure S9: SEM/EDS elemental distribution maps of the La_1.7_Ca_0.3_Ni_0.8_Cu_0.2_O_4+δ_ electrode surface (T_S_ = 1000 °C); Figure S10: SEM/EDS elemental distribution maps of the La_1.7_Ca_0.3_Ni_0.6_Cu_0.4_O_4+δ_ electrode surface (T_S_ = 1000 °C); Figure S11: Influence of electrode sintering temperature on the polarization resistance of La_1.7_Ca_0.3_Ni_1−y_Cu_y_O_4+δ_ electrodes in contact with SDC solid electrolyte; Table S1: Impurity content and specific surface area of the La_1.7_Ca_0.3_Ni_1−y_Cu_y_O_4+δ_ powders after the two-stage synthesis with intermediate and final milling; Table S2: Impurity content in the La_1.7_Ca_0.3_Ni_1−y_Cu_y_O_4+δ_ powders determined by SEM/EDS analysis; Table S3: Chemical composition of La_1.7_Ca_0.3_Ni_1−y_Cu_y_O_4+δ_ electrodes determined by SEM/EDS analysis of the electrode surface.
