# Supplementary material for: Development of La1.7Ca0.3Ni1−yCuyO4+δ Materials for Oxygen Permeation Membranes and Cathodes for Intermediate-Temperature Solid Oxide Fuel Cells
Source: Membranes (Basel). 2022 Dec 2;12(12):1222. doi: 10.3390/membranes12121222 (PMC9786882; doi:10.3390/membranes12121222)
Supplement: Supplementary file 1 [file membranes-12-01222-s001.zip › membranes-1999716-supplementary.pdf]

Table S1. Impurity content (according to the ICP-OES data) and specific surface area (according to the BET analysis data) of the $La_{1.7}Ca_{0.3}Ni_{1-y}Cu_yO_{4+\delta}$ powders after the two-stage synthesis with intermediate and final milling.

	**Impurity content, wt. %**		**S_{BET}, m^2·g^{-1}**
	**Si**	**Fe**	
**0.0**	0.41	0.38	1.16(2)
**0.2**	0.39	0.33	0.94(1)
**0.4**	0.42	0.41	0.97(2)

Table S2. Impurity content in the $La_{1.7}Ca_{0.3}Ni_{1-y}Cu_yO_{4+\delta}$ powders determined by SEM/EDS analysis.

**y**	**Fraction of impurities, wt. %**		**Fraction of impurities, at. %**	
	**Si**	**Fe**	**Si**	**Fe**
**0**	0.41	0.53	0.63	0.41
**0.2**	0.7	0.36	1.18	0.3
**0.4**	0.74	0.36	1.17	0.29

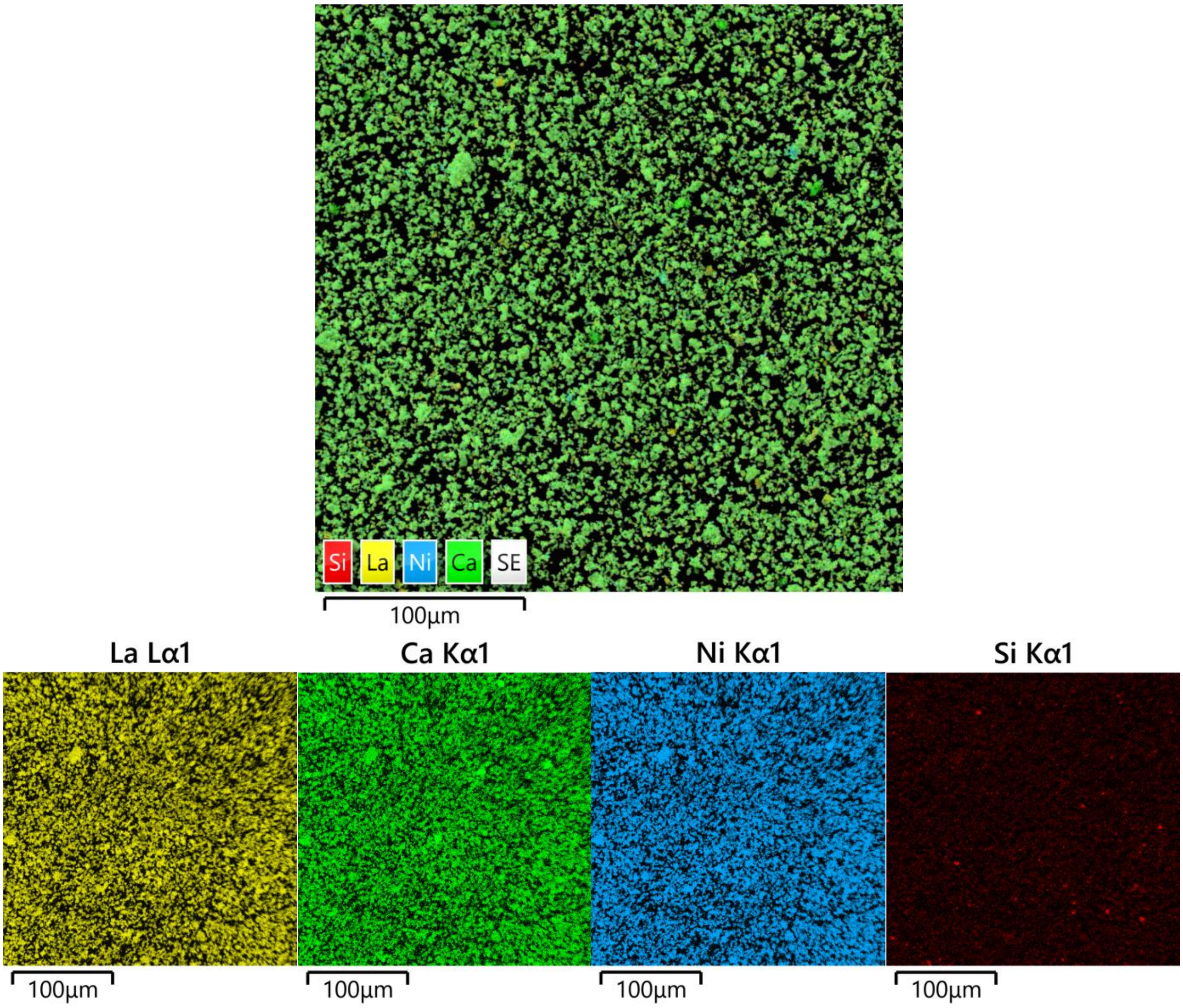

Figure S1. SEM/EDS elemental distribution maps of the $La_{1.7}Ca_{0.3}NiO_{4+\delta}$ powder

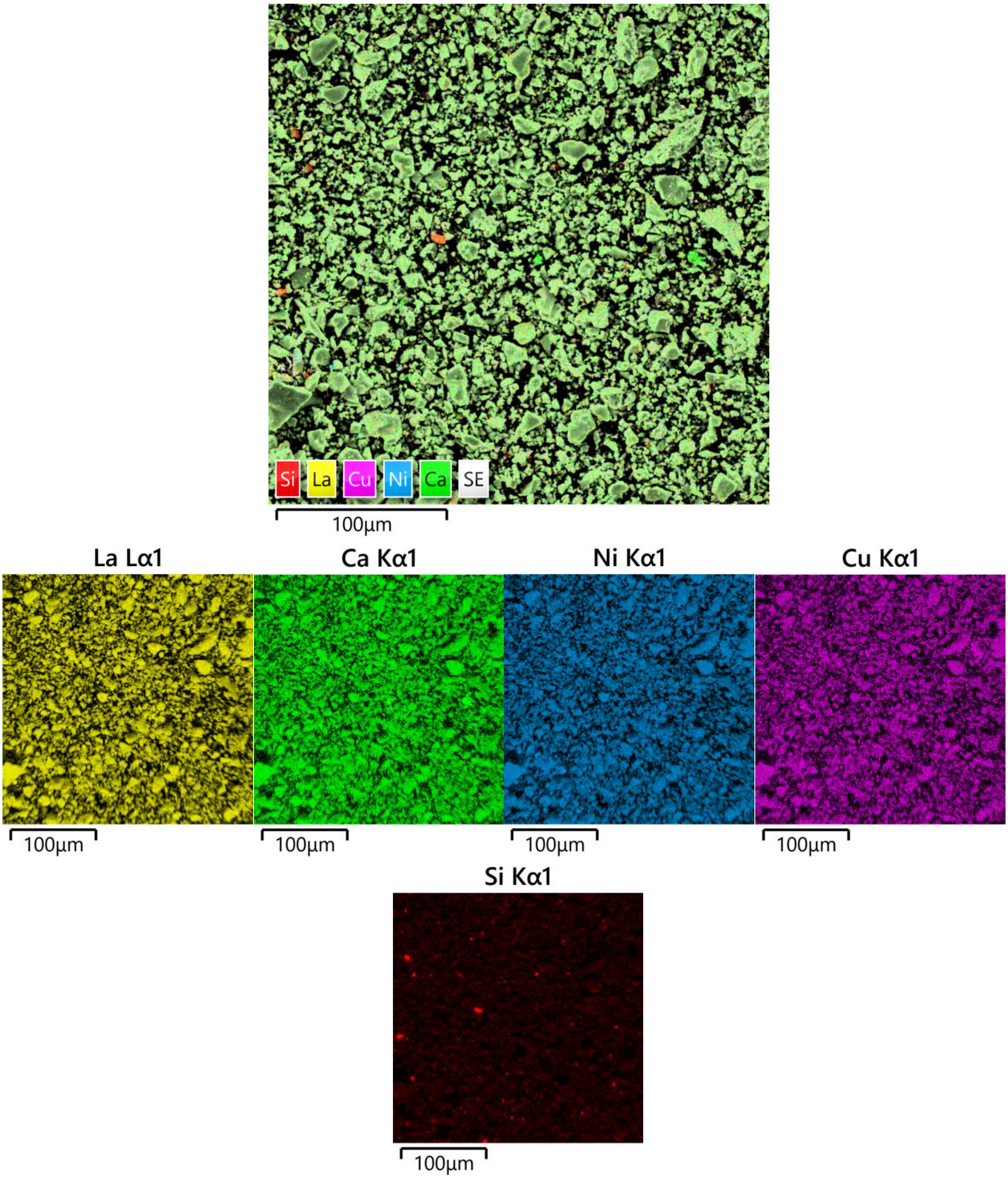

Figure S2. SEM/EDS elemental distribution maps of the $La_{1.7}Ca_{0.3}Ni_{0.8}Cu_{0.2}O_{4+\delta}$ powder

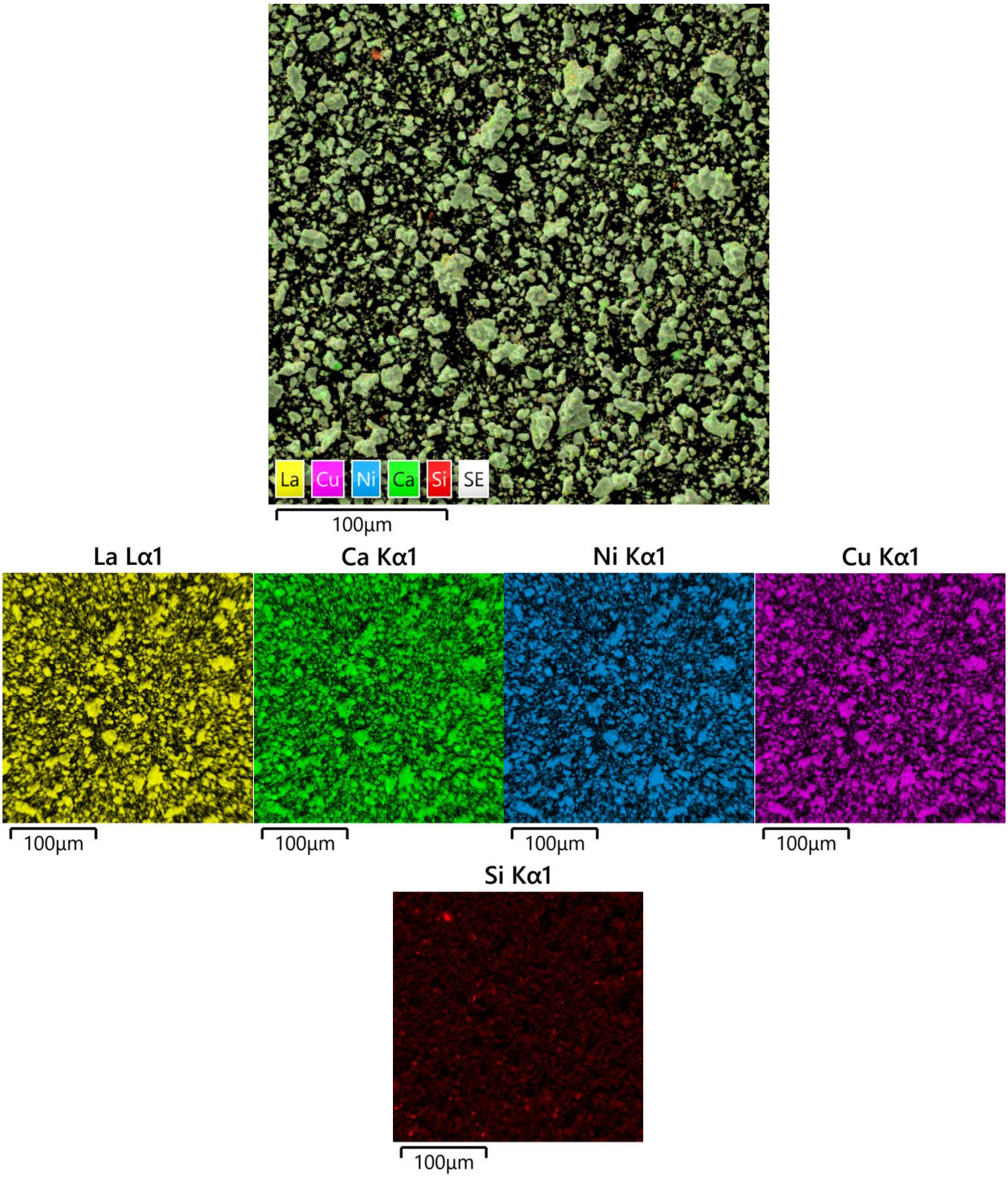

Figure S3. SEM/EDS elemental distribution maps of the $La_{1.7}Ca_{0.3}Ni_{0.6}Cu_{0.4}O_{4+\delta}$ powder

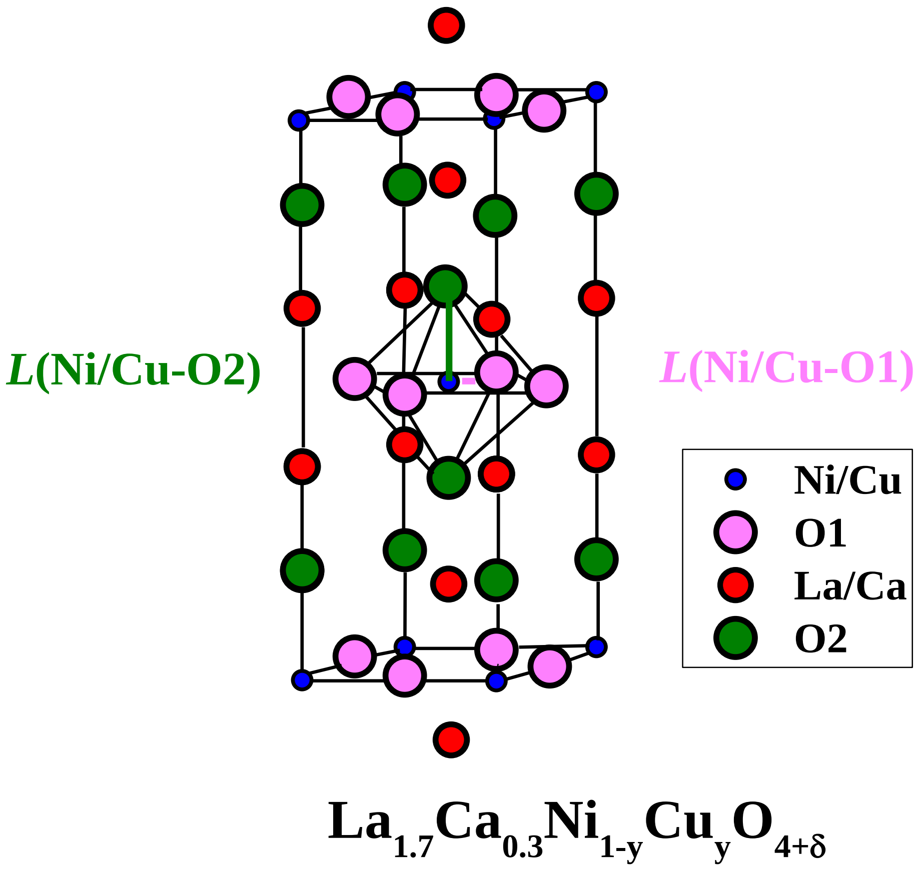

Figure S4. Crystal structure of $La_{1.7}Ca_{0.3}Ni_{1-y}Cu_{y}O_{4+\delta}$.

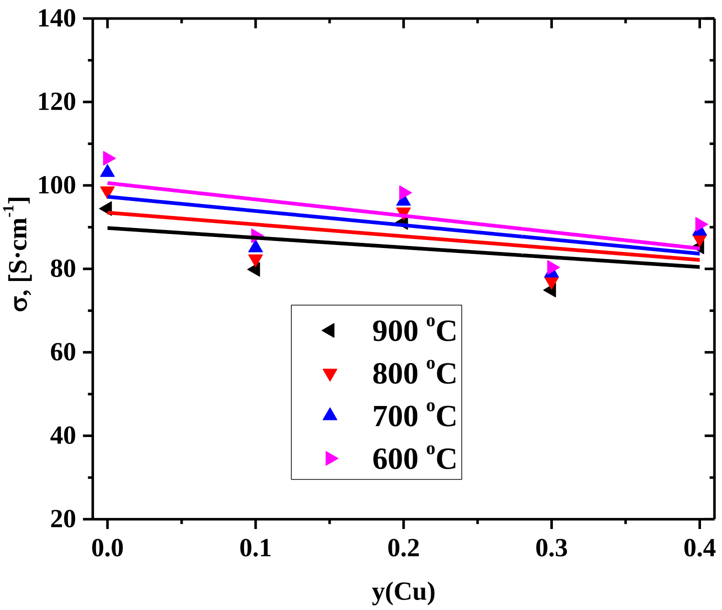

Figure S5. Concentration dependencies of the total conductivity of the $La_{1.7}Ca_{0.3}Ni_{1y}Cu_{y}O_{4+\delta}$ compact samples collected in air.

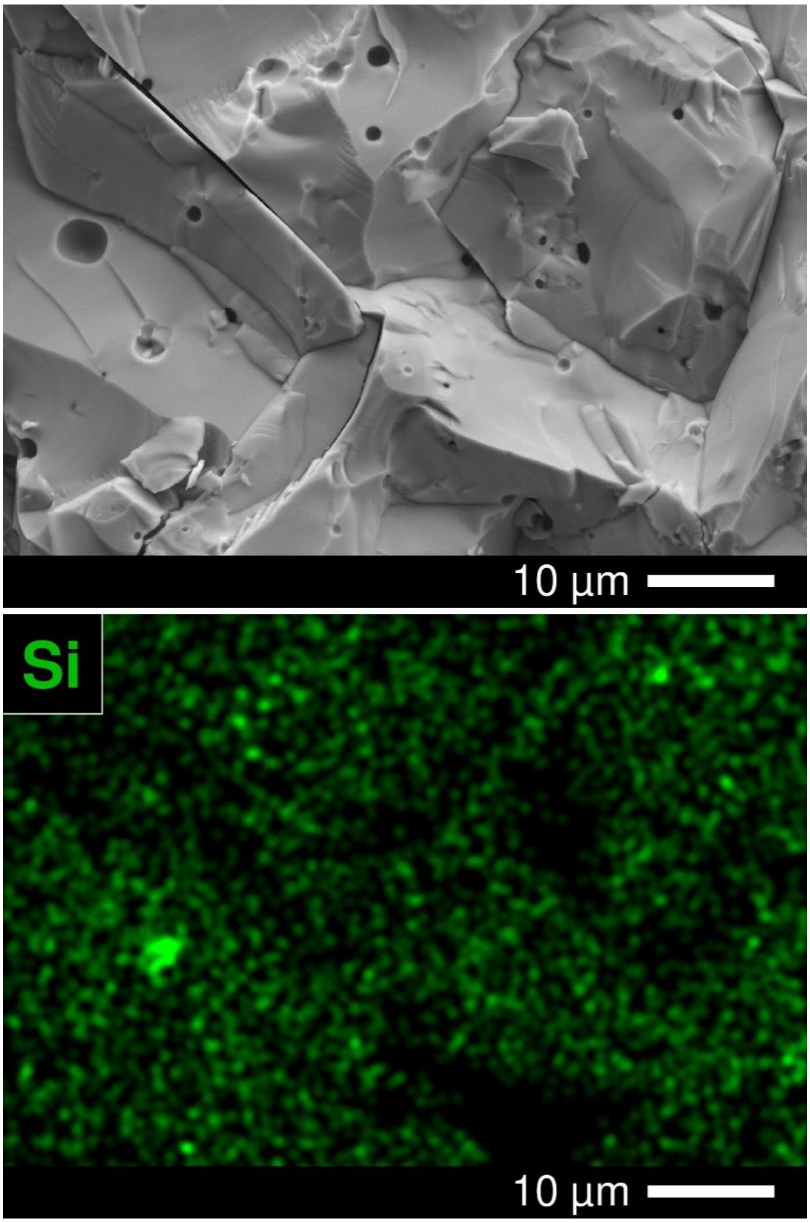

Figure S6. SEM/EDS: fractured surface of $La_{1.7}Ca_{0.3}Ni_{0.8}Cu_{0.2}O_{4+\delta}$ ceramics sintered at 1380 °C.

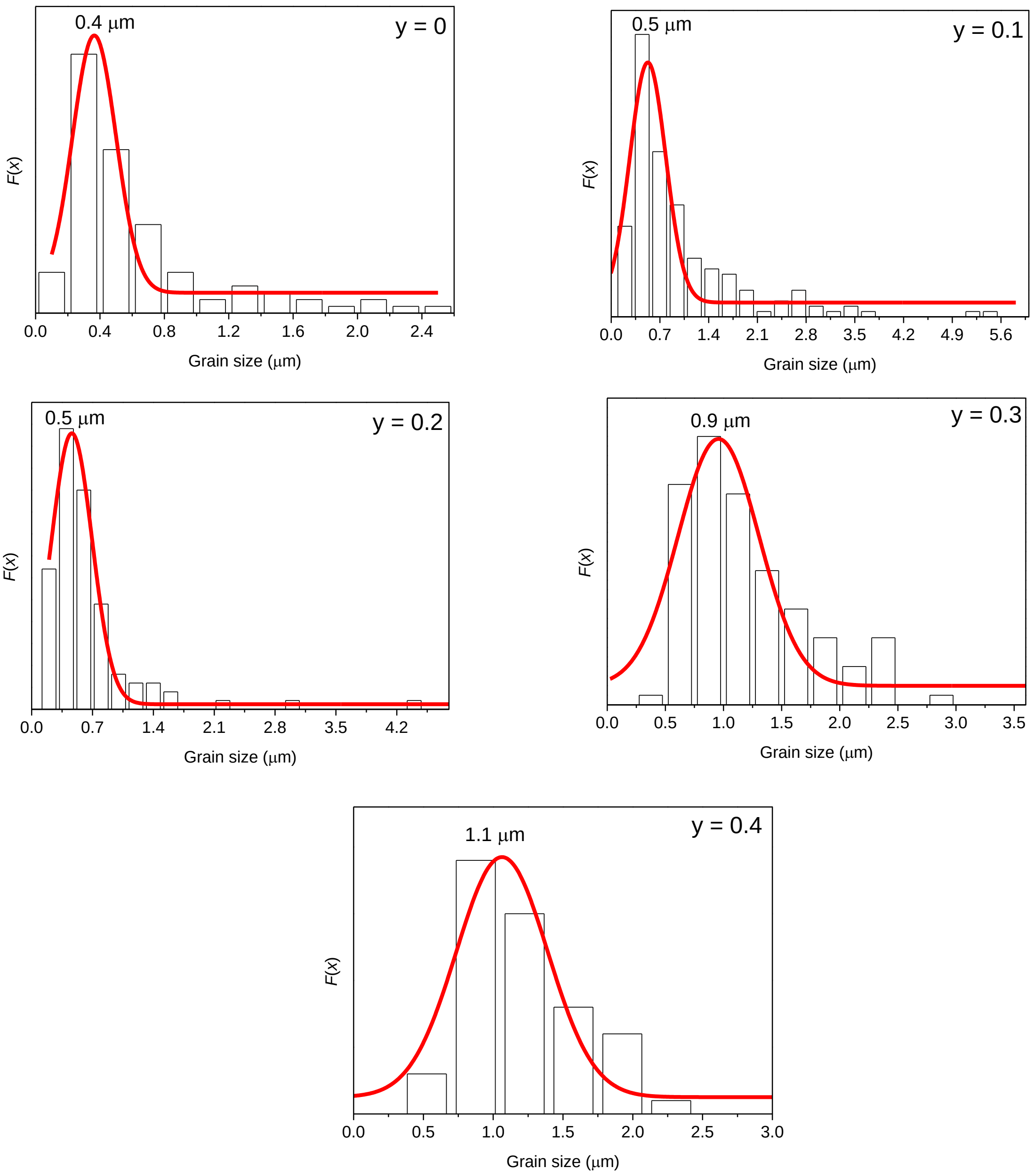

Figure S7. Particle size distribution in the $La_{1.7}Ca_{0.3}Ni_{1-y}Cu_yO_{4+\delta}$ electrodes based on the SEM data.

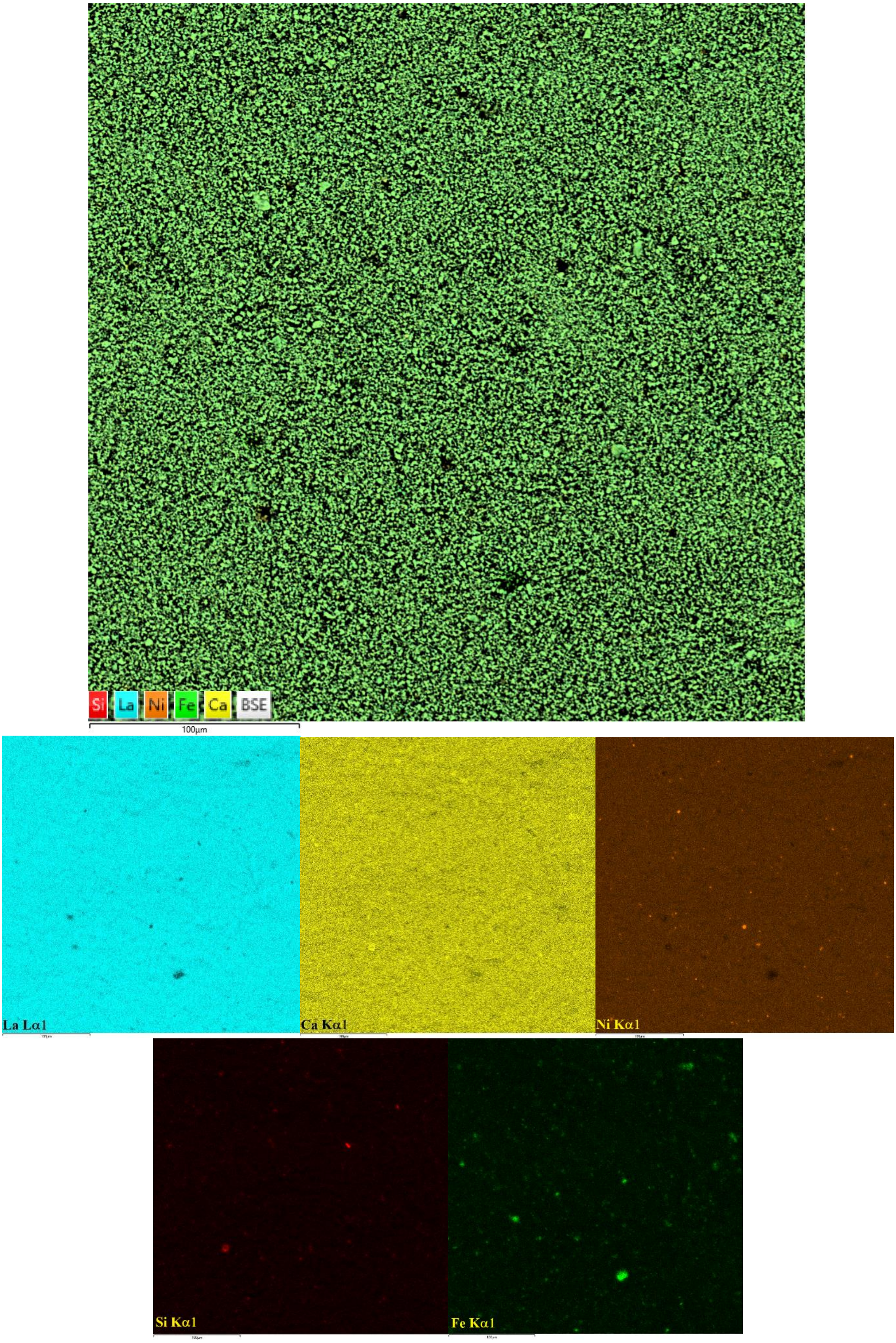

Figure S8. SEM/EDS elemental distribution maps of the $La_{1.7}Ca_{0.3}NiO_{4+\delta}$ electrode surface (T_S = 1000 °C).

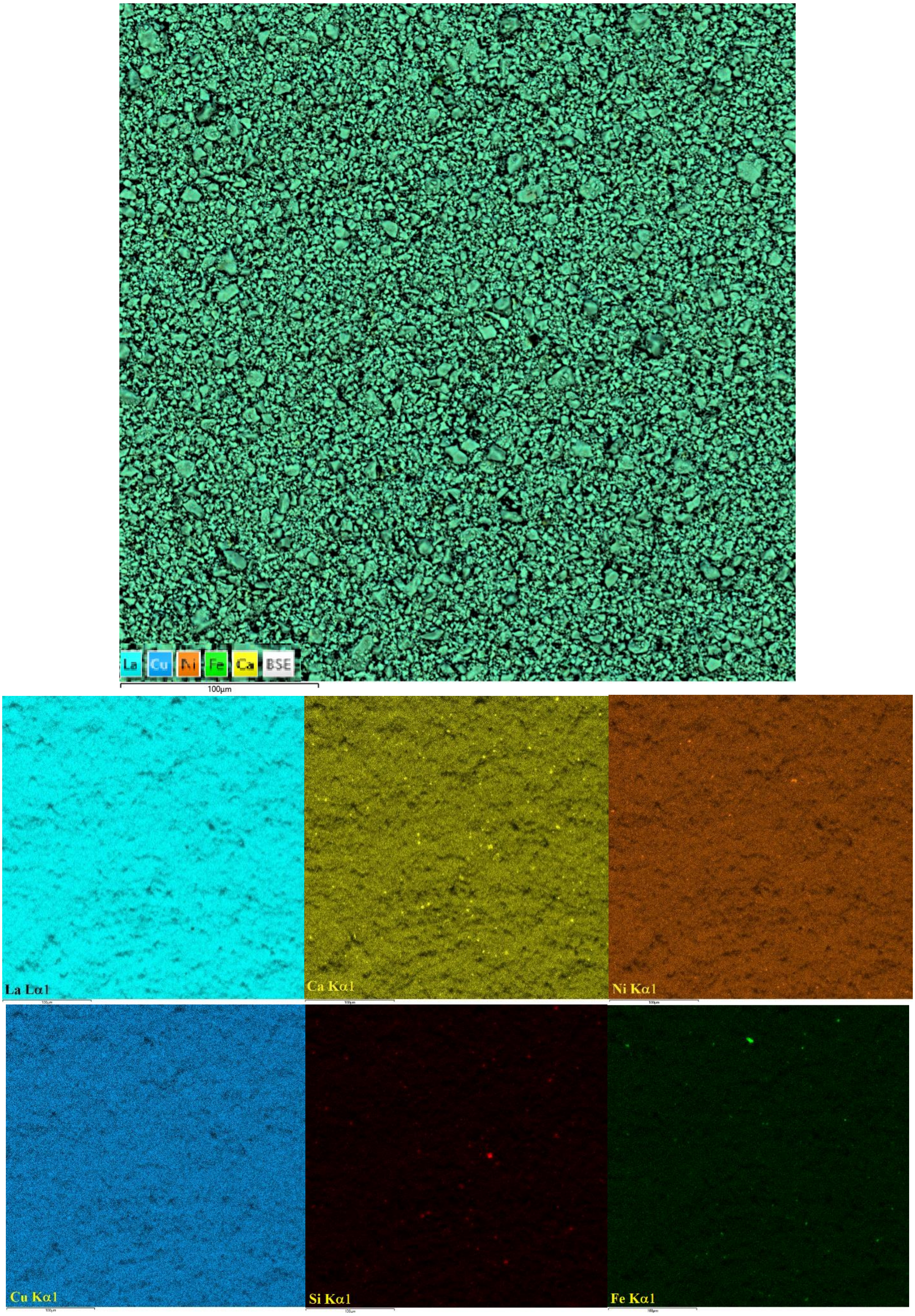

Figure S9. SEM/EDS elemental distribution maps of the $La_{1.7}Ca_{0.3}Ni_{0.8}Cu_{0.2}O_{4+\delta}$ electrode surface (T_S = 1000 °C).

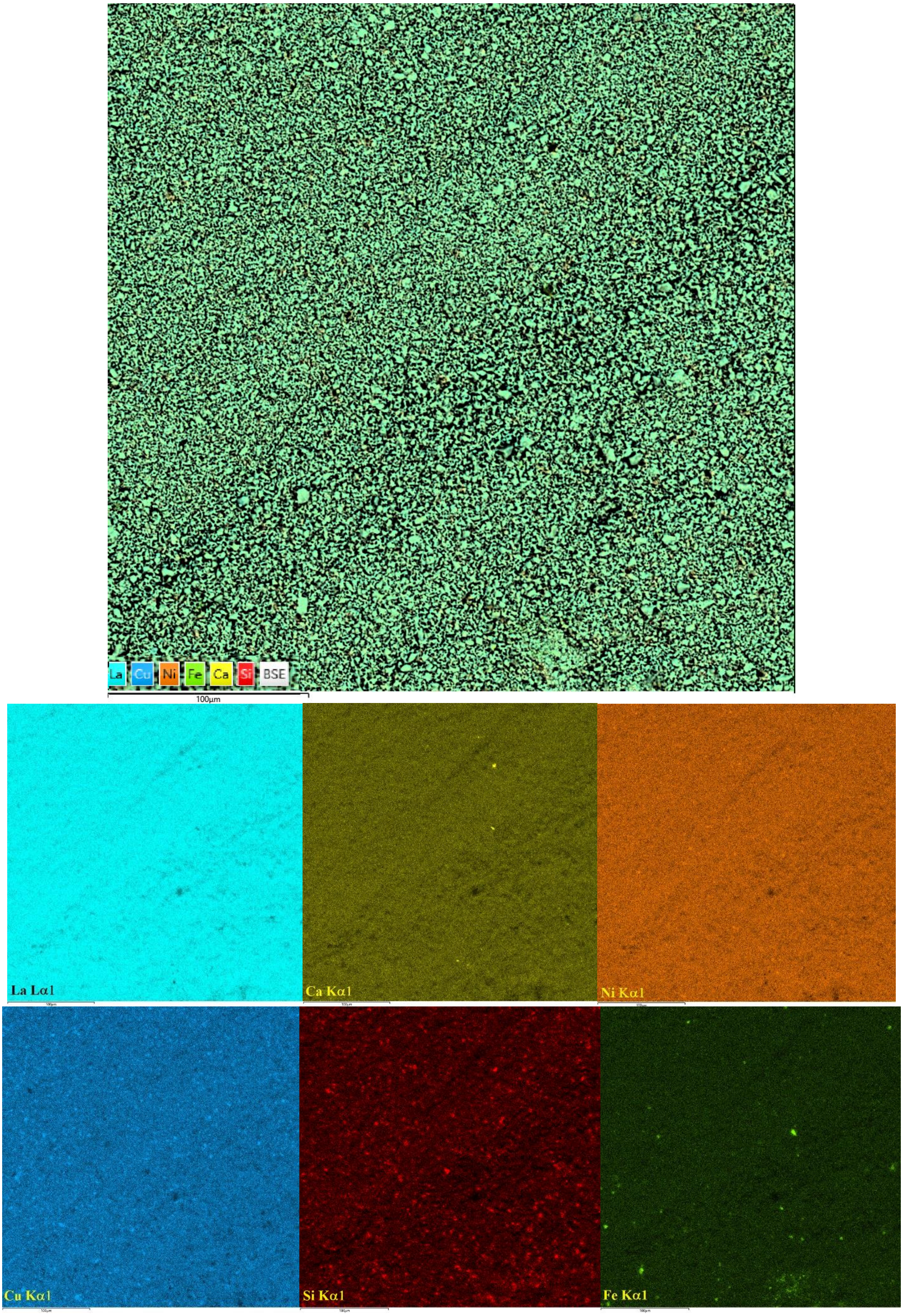

Figure S10. SEM/EDS elemental distribution maps of the $La_{1.7}Ca_{0.3}Ni_{0.6}Cu_{0.4}O_{4+\delta}$ electrode surface (T_S = 1000 °C).

Table S3. Chemical composition of $La_{1.7}Ca_{0.3}Ni_{1-y}Cu_yO_{4+\delta}$ electrodes determined by SEM/EDS analysis of the electrode surface.

y	Fractions of metal cations, at. %							La:Ca:Ni:Cu
	La	Ca	Ni	Cu	Si	Fe	Pt	
0	54.30	9.39	32.07	-	2.00	1.11	1.13	1.69:0.29:1.00:0
0.2	54.00	9.54	26.09	6.26	2.45	0.80	0.87	1.66:0.29:0.80:0.19
0.4	53.55	9.33	19.21	12.68	3.46	1.03	0.75	1.67:0.29:0.60:0.40

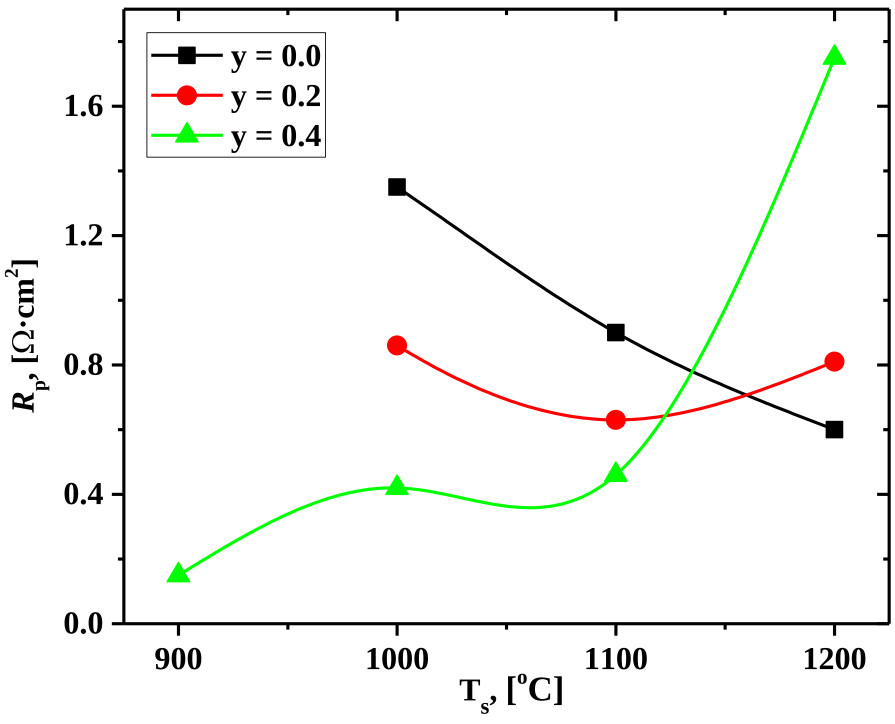

Figure S11. Influence of electrode sintering temperature on the polarization resistance of $La_{1.7}Ca_{0.3}Ni_{1-y}Cu_yO_{4+\delta}$ electrodes in contact withSDC solid electrolyte.
